# Contrasting patterns of nucleotide diversity for four conifers of Alpine European forests

**DOI:** 10.1111/j.1752-4571.2012.00256.x

**Published:** 2012-11

**Authors:** Elena Mosca, Andrew J Eckert, John D Liechty, Jill L Wegrzyn, Nicola La Porta, Giovanni G Vendramin, David B Neale

**Affiliations:** 1 Department of Plant Sciences, University of California at DavisDavis, CA, USA; 2 IASMA Research and Innovation Centre, Fondazione Edmund Mach, S. Michele all’AdigeItaly; 3 Department of Biology, Virginia Commonwealth UniversityRichmond, VA, USA; 4 Consiglio Nazionale delle Ricerche Plant Genetics InstituteSesto Fiorentino (Firenze), Italy; 5 Center for Population Biology, University of California at DavisDavis, CA, USA

**Keywords:** candidate gene, neutrality tests, nucleotide diversity, single nucleotide polymorphisms

## Abstract

A candidate gene approach was used to identify levels of nucleotide diversity and to identify genes departing from neutral expectations in coniferous species of the Alpine European forest. Twelve samples were collected from four species that dominate montane and subalpine forests throughout Europe: *Abies alba* Mill, *Larix decidua* Mill, *Pinus cembra* L., and *Pinus mugo* Turra. A total of 800 genes, originally resequenced in *Pinus taeda* L., were resequenced across 12 independent trees for each of the four species. Genes were assigned to two categories, candidate and control, defined through homology-based searches to *Arabidopsis.* Estimates of nucleotide diversity per site varied greatly between polymorphic candidate genes (range: 0.0004–0.1295) and among species (range: 0.0024–0.0082), but were within the previously established ranges for conifers. Tests of neutrality using stringent significance thresholds, performed under the standard neutral model, revealed one to seven outlier loci for each species. Some of these outliers encode proteins that are involved with plant stress responses and form the basis for further evolutionary enquiries.

## Introduction

Subalpine landscapes in the Northern Hemisphere are dominated by coniferous tree species. These ecosystems are sensitive to climate change (Cannone et al. 2008; Theurillat and Guisan 2001). The ability of conifer populations to track climate optima through genetic adaptation will be difficult given the rapidity of climate change (Pautasso 2009), thus raising questions about the relative roles of *in situ* versus *ex situ* conservation efforts (Aitken et al. 2008). Genetic diversity is fundamentally important to both approaches, because the continued adaptability, health, and long-term productivity of trees are driven by genetic diversity (Schaberg et al. 2008). Conservation efforts for tree populations, therefore, will benefit greatly from descriptions of genetic diversity. Here, we estimate levels of nucleotide diversity for four dominant tree species in the European forests –*Abies alba* Mill, *Larix decidua* Mill, *Pinus cembra* L., and *Pinus mugo* Turra – using DNA sequence data from 800 genes. These species are the main components of the subalpine forest ecosystem of the Alps, being found along an altitudinal gradient from 500 to over 2000 m.

Trees growing at alpine and subalpine zones may be under strong abiotic stresses and often display phenotypic responses to stressful environments (Gamache and Payette 2004). In high mountain areas, both species distribution and the forest composition are shaped by climatic conditions (Bonan 2008; Grace 2002). An increase in temperature could shift species ranges to higher elevations, as has been shown for *Pinus peuce* Griseb. (Meshinev et al. 2000), *Fagus sylvatica* L. (Peñuelas and Boada 2003), and *P. mugo* (Camarero et al. 2005). Conversely, climate change has led to a complex shift of species elevational distributions in the Sierra Nevada Mountains of California (Crimmins et al. 2011).

Genetic studies will help to better understand the complexity of adaptation, by demonstrating the role of natural selection in this process. The importance of natural selection has traditionally been demonstrated in trees using common garden experiments (Eveno et al. 2008; González-Martínez et al. 2006). The timing of annual growth (phenology) and response to abiotic stresses (e.g., temperature, moisture) are primary target traits for adaptive studies (Garnier-Géré and Ades 2001; Aitken and Hannerz 2001). However, the genes underlying these quantitative traits, and the segregating polymorphism within these genes, remain unknown. Single nucleotide polymorphisms (SNPs) within candidate genes for complex adaptive traits provide informative markers for studies of natural selection (Neale and Savolainen 2004). Loci with unusually high or low levels of variation (outlier loci) may be affected by selective forces (Luikart et al. 2003) and can be detected by outlier analysis (Kelley et al. 2006). Patterns of diversity and divergence can be predicted for simple null models, so that values observed for sampled genes can be assessed for their consistency to expectations derived from these null models.

The outlier approach has been used in several forest trees, including species of *Quercus* (Scotti-Saintagne et al. 2004), *Pinus* (González-Martínez et al. 2006), *Pseudotsuga* (Eckert et al. 2009), and *Picea* (Namroud et al. 2008; Holliday et al. 2010), to detect genes showing contrasting patterns of variation. In particular, several studies have focused on specific genes involved in the adaptation to cold (Eckert et al. 2009; Wachowiak et al. 2009) and to drought tolerance (Pyhäjärvi et al. 2007; Eveno et al. 2008; González-Martínez et al. 2008; Grivet et al. 2009, 2011), thus enabling the application of a population genetic approach to the study of adaptation.

The specific aims of this study were (i) to discover SNPs sampled from natural populations of each species; (ii) to estimate nucleotide diversity in the four target species; (iii) to identify among a large set of putative candidate genes those that may be under natural selection; and (iv) to determine whether the genes found to be potentially under selection were common among the four species.

## Material and methods

### Species

*Abies alba* is primarily a mountain species, distributed throughout western, central, and southern Europe (Farjon 1990). The natural range for this species is patchy, as a result of several migratory pathways following the end of the last ice age (Liepelt et al. 2009). *Larix decidua* is naturally distributed in both central and eastern Europe (Rubner 1953). It occurs in the high mountains of central Europe between 1000 and 2200 m, while in Central Alps, it can be found even at higher elevation (Farjon 1990). *Pinus cembra* and *P. mugo* are both pioneer species that form pure or mixed stands; above timberline, *P. cembra* is usually present as solitary individuals, while *P. mugo* may form pure and dense stands. *Pinus mugo* is phenotypically variable with a complex classification (Monteleone et al. 2006), mainly due to its high morphological variability in growth habit (Christensen 1987; Wachowiak and Prus-Głowacki 2008). It is native to the mountains in central and southern Europe. *Pinus cembra* is a glacial relict that has survived in the high European mountains (Höhn et al. 2005). Its fragmented range is mainly due to postglacial competition with *Picea abies* Karst (Höhn et al. 2009).

### Sampling

**Figure 1:**
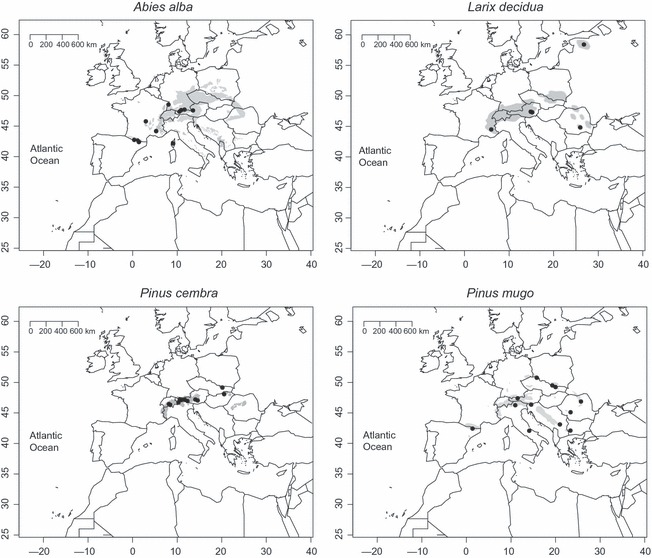
Sampling locations across species distribution in Europe.

Sampled trees were selected to cover the natural distribution of each species. Several seeds were collected from one individual at each of the 12 natural populations per species located in European Mountains (Fig. 1; Table S1). For each sampled tree, the latitude and longitude positions were recorded.

### Identification of candidate genes

Sequencing primers used in this study resulted from previous research on *P. taeda* L. (cf. http://dendrome.ucdavis.edu/NealeLab/adept2/), where a set of 7535 primer-pairs was developed for Sanger resequencing (Eckert et al. 2010a), using clustered ESTs (Expressed Sequence Tags). A subset of 800 primer pairs (Data S1) that successfully amplified and resequenced in other species within the *Pinaceae* (cf. http://dendrome.ucdavis.edu/NealeLab/crsp/) were used in the present study. The process of data generation is outlined in Fig. S1. A total of 698 genes of the 800 attempted had sequence data for at least one species following the removal of duplicate sequences and organellar sequences. To identify the potential protein function of the 698 genes, BLAST, BLASTp, and tBLASTx analyses were performed in the NCBI (http://www.ncbi.nlm.nih.gov/) and TAIR (http://www.arabidopsis.org/) databases, using published sequences of *Arabidopsis thaliana*. When possible, previous studies on gene expression in other conifers were also taken into account when defining candidate genes (Eveno et al. 2008). From the unique gene data set, a subset of 430 candidate genes was identified based on their biological function in Arabidopsis. The remaining 268 genes were used as genomic controls to contrast with estimates of nucleotide diversity, divergence, and outlier detection in candidate genes (Devlin et al. 2000). After removing low-quality data, the data set consists of 246 candidate genes and 156 control genes in total. The exact number of genes for each species is reported in Fig S1.

### DNA isolation and resequencing

After seed germination, DNA was isolated from the haploid megagametophytes using Qiagen Plant Mini kits (Qiagen, Valencia, CA, USA) and stored at −80°C. Each DNA extraction consisted of a single megagametophyte. The DNA was amplified subsequently with RepliG kit (Qiagen) following the manufacturer’s protocol. The amplified product was purified and sequenced directly following standard protocols (Eckert et al. 2010b). The 800 amplicons were resequenced using Sanger sequencing methods by Agencourt Bioscience Corporation (Beverly, MA, USA).

### Sequence analysis

A customized pipeline, PineSAP (Wegrzyn et al. 2009), was used to generate sequence alignments and to identify SNPs. A customized Perl script (https://dendrome.ucdavis.edu/TGPlone/research-projects/ace-sap/) was used subsequently to identify sequencing primers in each alignment and to mask bases outside the sequence interval defined by these primers whose signal would be due to mispriming. As megagametophyte samples were not pooled, each DNA sample is derived from a single meiotic event, so it is not expected to see problems associated with Sanger sequencing in diploid tissue (e.g., calling heterozygous SNPs or phasing owing to heterozygous indels). This also allowed rejecting samples that contained potentially paralogous sequence by detecting the presence of secondary peaks in the chromatograms. The quality of base calls, especially those associated with SNPs, was confirmed visually using CONSED (Gordon et al. 1998). The sequence alignments were subsequently aligned with *P. taeda*, which was used as an outgroup for each species separately using the profile-profile option in MUSCLE (Edgar 2004). Manual adjustments to these alignments were performed using Se-Al version 2.0a9 when necessary (Sequence Alignment Editor version 2.0, Rambaut 1996–2002). All sequences were deposited in GenBank (JQ440374-JQ445205).

Sites within genetic loci were annotated manually by first aligning sequences with the *P. taeda* ESTs to identify introns. A tBLASTx analysis was subsequently performed against the refseqRNA database for *Arabidopsis* to identify the putative coding intervals for each gene. Finally, BLASTp analyses against *Arabidopsis thaliana* gene models used to derive coding intervals were used to verify delineation of coding intervals and frame for each locus. These annotations were successfully performed for 80% of the candidate genes and 50% of the control genes (Data S4).

### Nucleotide diversity and divergence

Aligned sequences were analyzed with the DNA Sequence Analysis and Manipulation (DnaSAM) program (Eckert et al. 2010b). Sites with missing data, with indels, or that violated the infinite sites mutational model were not included in the analysis. For each gene, nucleotide diversity per site was estimated with θ_π_ (Tajima 1983; Nei 1987), the average pairwise difference between sequences, and θ_w_ (Watterson 1975), which is based on the number of segregating sites. Pairwise divergence (*D*_xy_) was computed between each species and *P. taeda*, which was represented as a majority rules consensus sequence. Based on the subset of the annotated genes, nucleotide diversity at synonymous and nonsynonymous sites was calculated with the polydNdS program in the analysis package of libsequence C++ library (Thornton 2003). Using gestimator from the same package, divergence per site was calculated for different categories of sites. Differences between species-specific means for diversity and divergence estimates were evaluated through bootstrapping across loci (*n* = 10 000 replicates). The 95% bootstrap confidence intervals were compared to assess differences between means. All statistical analyses were performed using the boot package in R (R Development Core Team 2007).

### Neutrality tests

To search for patterns of diversity that were not consistent with the standard neutral model (SNM), four neutrality statistics were computed for each species: Ewens–Watterson *F* (Ewens 1972), Tajima’s *D* (Tajima 1989), Fay and Wu’s normalized *H* (Fay and Wu 2000), and Kelly’s Zns (Kelly 1997). Each statistic for each species was tested against the SNM model using coalescent simulations (*n* = 10,000). Coalescent simulations were conducted using the ms program (Hudson 2002) as implemented using DnaSAM to estimate *P*-values under the SNM.

The multidimensional DHEW test that combines three neutrality tests – Tajima’s *D*, Fay and Wu’s normalized *H*, and Ewens–Watterson *F* (Zeng et al. 2006, 2007) – was applied to find the loci that departed from neutrality. The nominal threshold for the calculation of multidimensional *P*-values was set at *a* = 0.0001, from which an adjusted nominal significance level (P*) was estimated from 50 000 coalescent simulations, conditional on the SNM and the observed value of θ_π_. All multidimensional tests were conducted using DnaSAM. The genes with only one SNP were removed from the analysis to avoid false-positive outliers owing to their low nucleotide diversity.

## Results

### Nucleotide diversity and divergence in all genes

The average gene fragment length for all species was between 380 bp (±117) and 401 bp (±109) (Table 1), and average number of sequences per gene ranged from three (*L. decidua*) to six (*P. cembra*). On average, nearly half of the candidate genes were polymorphic in all species, with the exception of *P. mugo*, where more than 70% of the genes displayed variation. Among the control genes, *P. mugo* was also the most polymorphic, but for this set of genes, *L. decidua* and *P. cembra* had less than 50% polymorphic genes.

**Table 1:** Sequencing summary statistics for the candidate genes and the control genes for each species.

	Candidate				Control			
	
	*Abies alba*	*Larix decidua*	*Pinus cembra*	*Pinus mugo*	*A. alba*	*L. decidua*	*P. cembra*	*P. mugo*
Total genes	70	61	171	190	32	35	109	120
Length mean (bp)	**380** ± 117	**389** ± 123	**398** ± 117	**386** ± 119	**389** ± 105	**401** ± 109	**379** ± 106	**381** ± 106
No. of samples	**5** ± 3	**3** ± 1	**6** ± 3	**4** ± 2	**5** ± 3	**4** ± 2	**6** ± 3	**5** ± 2
Polymorphic genes (%)	54.29	50.82	45.03	73.16	56.25	37.14	34.26	73.33
Total no. of SNPs	197	219	284	900	131	88	90	487
No. of SNPs per gene	3 ± 6	4 ± 9	2 ± 4	5 ± 9	4 ± 9	3 ± 7	1 ± 2	4 ± 7
SNP frequency	135	108	239	81	95	159	459	94
Watterson’s θ	**0.0059** 0.014	**0.0077** 0.020	**0.0025** 0.007	**0.0082** 0.039	**0.0064** 0.014	**0.0044** 0.012	**0.0013** 0.006	**0.0069** 0.013
Nucleotide diversity (π)	**0.0059** 0.0134	**0.0078** 0.020	**0.0024** 0.007	**0.0081** 0.079	**0.0061** 0.013	**0.0047** 0.014	**0.0013** 0.006	**0.0067** 0.013
Divergence	**0.0873** 0.0326	**0.0829** 0.031	**0.0378** 0.017	**0.0157** 0.013	**0.0820** 0.039	**0.0734** 0.036	**0.0373** 0.025	**0.0159** 0.012

Mean values are reported in boldface type with their standard deviation.

**Figure 2:**
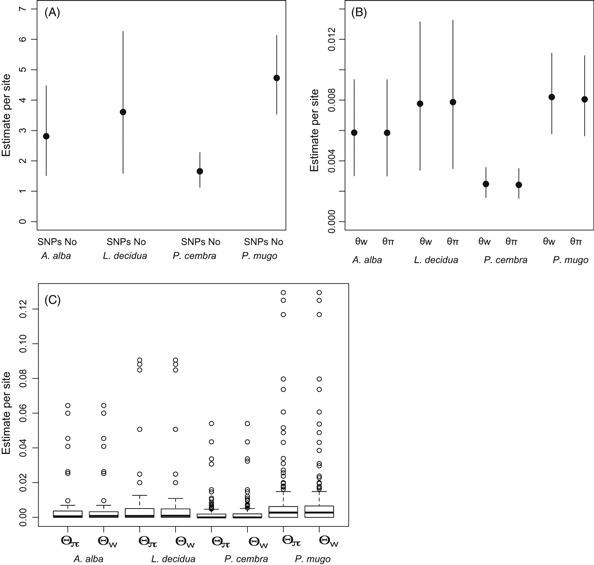
Mean values of (A) number of SNPs and (B) nucleotide diversity (θ_w_, θ_π_) with the bootstrapped 95% confidence intervals and (C) distribution of nucleotide diversity in candidate genes for the different species. The box shows the upper and lower quartiles, while the line represents the median of the sample.

The total number of SNPs ranged from 197 in *A. alba* to 900 in *P. mugo* in the candidate genes, while in the control genes, it ranged from 88 in *L. decidua* to 487 in *P. mugo* (Table 1). The total number of SNPs per gene ranged from 0 to a high of 56 that was found in *L. decidua* (Fig. S4), and the average number of SNPs per gene ranged from 2 to 5 (Table 1). However, for the candidate genes, the only significant pairwise difference in the mean number of SNPs was between *P. cembra* and *P. mugo*, supported by the bootstrapped 95% confidence intervals (Fig. 2A).

Among candidate genes, average estimates of Watterson’s θ ranged from 0.0025 in *P. cembra* to 0.0082 in *P. mugo* (Table 1). With the exception of *A. alba*, the average estimates of Watterson’s θ were lower in the control genes than in candidate genes (Table 1). The two average estimates of diversity (θ_π_ vs θ_w_) did not differ within species (Fig. 2B). The distribution of θ_π_ and θ_w_ varied widely in the candidate genes (Fig. 2C) and the control genes (Fig. S2A). Nucleotide diversity estimates varied significantly among species, supported by the bootstrapped 95% confidence intervals (Fig. 2B). However, for the candidate genes, the only significant pairwise difference in nucleotide diversity estimates was between *P. cembra* and *P. mugo*, supported by the bootstrapped 95% confidence intervals (Fig. 2B).

Estimates of nucleotide divergence for candidate and control genes varied among species (Table 1) but were consistent with their phylogenetic position relative to *P. taeda* (Gernandt et al. 2008; Eckert and Hall 2006; Willyard et al. 2007). *Abies alba* showed the highest divergence from *P. taeda*. According to a conservative fossil calibration (Gernandt et al. 2008), the separation between the two subfamilies (*Abietoideas* and *Pinoidea*) occurred around 136 million years ago (mya). A more recent separation within the subfamily *Pinoideae* determined the formation of genus *Larix* (around 133 mya). Moreover, the two pines had different estimates of divergence. This result is consistent with their membership in the two subgenera of *Pinus*; *P. cembra* belongs to the subgenus *Strobus*, while both *P. mugo* and *P. taeda* belong to subgenus *Pinus*.

### Nucleotide diversity and divergence in the annotated candidate genes

The percentage of silent sites ranged from 35% in *L. decidua* to 40% in *P. cembra* (Table 2). The number of segregating sites in all regions ranged from 80 in *L. decidua* to 574 in *P. mugo*, whereas the percentage of segregating sites in nonsynonymous regions varied from 29% in *P. cembra* to 43% in *A. alba*. In all species, the majority of the SNPs were silent with values ranging from 57% in *A. alba* to 71% in *P. cembra*. On average, two nonsynonymous SNPs per gene were found in *P. mugo* (range: 0–46), one in *A. alba* (range: 0–20) and 0.5 SNPs per gene in both *L. decidua* (range: 0–16) and *P. cembra* (range: 0–12).

**Table 2:** Levels of nucleotide polymorphism in the annotated candidate genes

Species	Parameters	All^a^	N-coding^a^	N-Syn^a^	Syn^a^	All silent^a^
*Abies alba*	Sites^b^	**23 299**	**4362**	**14 650**	**4005**	**8367**
			18.72%	62.88%	17.19%	35.91%
	Segregating sites^b^	**146**	**23**	**63**	**60**	**83**
			15.75%	43.15%	41.10%	56.85%
	Watterson’s θ^c^	**0.0050**	**0.0027**	**0.0034**	**0.0097**	
		0.0133	0.0129	0.0097	0.0299	
	Nucleotide diversity (π)^c^	**0.0050**	**0.0027**	**0.0034**	**0.0097**	
		0.0133	0.0013	0.0097	0.0294	
*Larix decidua*	Sites^b^	**20 265**	**3571**	**12 862**	**3559**	**7130**
			17.62%	63.47%	17.56%	35.18%
	Segregating sites^b^	**80**	**14**	**26**	**40**	**54**
			17.50%	32.50%	50.00%	67.50%
	Watterson’s θ^c^	**0.0032**	**0.0008**	**0.0017**	**0.0087**	
		0.0075	0.0021	0.0058	0.0206	
	Nucleotide diversity (π)^c^	**0.0033**	**0.0009**	**0.0017**	**0.0088**	
		0.0075	0.0023	0.0058	0.0207	
*Pinus cembra*	Sites^b^	**57 152**	**13 292**	**33 950**	**9343**	**22 635**
			23.26%	59.40%	16.35%	39.60%
	Segregating sites^b^	**222**	**69**	**64**	**89**	**158**
			31.08%	28.83%	40.09%	71.17%
	Watterson’s θ^c^	**0.0022**	**0.0013**	**0.0010**	**0.0049**	
		0.0057	0.0057	0.0035	0.0131	
	Nucleotide diversity (π)^c^	**0.0022**	**0.0013**	**0.0010**	**0.0049**	
		0.0057	0.0057	0.0034	0.0131	
*Pinus mugo*	Sites^b^	**62 260**	**12 541**	**38 447**	**10 693**	**23 234**
			20.14%	61.75%	17.17%	37.32%
	Segregating sites^b^	**574**	**123**	**234**	**217**	**340**
			21.43%	40.77%	37.80%	59.23%
	Watterson’s θ^c^	**0.0057**	**0.0028**	**0.0034**	**0.0112**	
		0.0129	0.0068	0.0102	0.0261	
	Nucleotide diversity (π)^c^	**0.0055**	**0.0028**	**0.0033**	**0.0108**	
		0.0128	0.0068	0.0101	0.0258	

a All, all sites; N-coding, noncoding sites; N-Syn, nonsynonymous sites; Syn, synonymous sites; All silent, all silent sites.

b Numbers are the total number of sites and the total number of segregating size.

c Bold numbers are the average across loci with their standard deviations in regular type.

**Figure 3:**
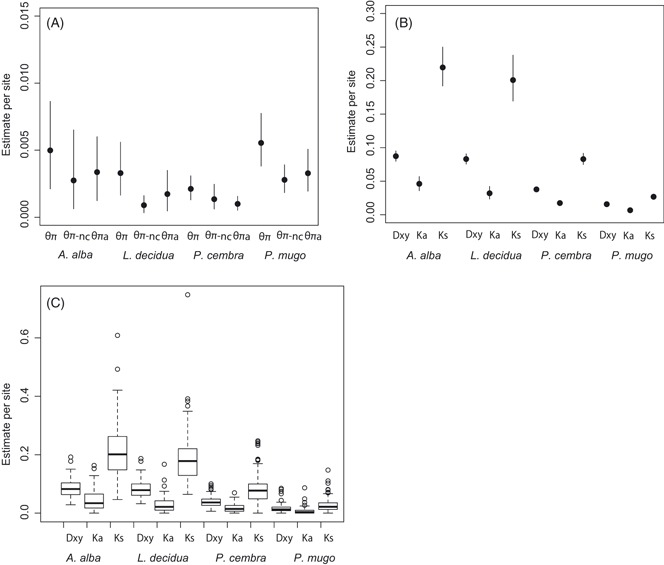
Mean values of (A) π for all sites for the annotated genes (θ_π_), at noncoding sites (θ_π-nc_), at nonsynonymous sites (θ_πa_) (B) divergence for all sites (*D*_xy_), divergence at nonsynonymous sites (*K*_a_), divergence at synonymous sites (*K*_s_), with the bootstrapped 95% confidence intervals and (C) distribution of divergence from loblolly pine across site categories in candidate genes. The box shows the upper and lower quartiles, while the line represents the median of the sample.

Average estimates of nucleotide diversity per site did not differ between θ_π_ and θ_w_; they varied among genes (Fig. S3A) and among species with values from θ_π_ = 0.0022 in *P. cembra* to θ_π_ = 0.0055 in *P. mugo* (Table 2). However, the only significant pairwise difference in the estimates of θ_π_ for all sites was between *P. cembra* and *P. mugo*, supported by the bootstrapped 95% confidence intervals (Fig. 3A). Within each species, average estimates of θ_π_ at noncoding sites were generally lower than the estimates of θ_π_ at nonsynonymous sites, with the exception of *P. cembra* (Fig. 3A). The ratio of the nonsynonymous to synonymous substitution rate is an indicator of selection. Neutral genes should have a ratio close to one. All polymorphic genes showed a ratio θ_πa_/θ_πs_ lower than 1.0 in *A. alba* and *P. cembra*, suggesting the presence of purifying selection, while one control gene had a ratio higher than 1.0 in *L. decidua*. In *P. mugo*, five candidate genes had ratios greater than one, with values falling in the range between 1.09 and 1.99 (Data S5).

The estimate of divergence from *P. taeda* in the annotated candidate genes varied among species supported by the bootstrapped 95% confidence intervals (Fig. 3B). The highest divergence calculated for all sites was found for *A. alba* and *L. decidua*, whose values were almost three times higher than what was found in *P. cembra*. Moreover, the average estimates of divergence varied within species among category of sites (Fig. 3B): divergence at synonymous sites (*K*_s_) was threefold greater than at nonsynonymous sites (*K*_a_). Within each species, estimates of divergence varied among genes (Fig. 3C). In general, estimates of divergence varied more in *A. alba* (range: 0.01476–0.02093) and *L. decidua* (range: 0.0063–0.1869) than in the two pines (range: 0.004–0.1622 in *P. cembra* and 0.0014–0.085 in *P. mugo*).

The majority of the candidate genes exhibited a *K*_a_/*K*_s_ ratio lower than 1.0 in all species, indicating the possible presence of purifying selection. One candidate gene (locus CL3007Contig1) showed a ratio higher than 1.0 in both *A. alba* and *L. decidua*. Four candidate genes in *P. cembra* (range: 1.10–1.83) and eight genes in *P. mugo* (range: 1.05–11.33) had a ratio greater than 1.0. In the latter species, the ratio ranged from 1.05 to 4.36; however, one gene showed an extremely high ratio (locus 0_4032, *K*_a_/*K*_s_ = 11.33) owing to both a lower *K*_a_ and a higher *K*_s_ compared to average estimates.

The percentage of silent sites in the annotated control genes ranged from 36.86% in *P. cembra* to 40.39% in *P. mugo* (Table S2). Average estimates of nucleotide diversity per site did not differ between θ_π_ and θ_w_; they varied among species (Fig. S2) and among genes (Fig. S3B) with values from θ_π_ = 0.0005 in *P. cembra* to θ_π_ = 0.0112 in *P. mugo* (Table S2).

### Neutrality tests

The results of individual candidate and control gene neutrality tests are presented in Data S3 and S6, and values varied enormously among genes. Average estimates of the neutrality test statistics reflect the differences in nucleotide diversity between candidate and control genes (Table S3). Among the candidate genes, Tajima’s *D* had a negative value on average in the majority of the species (*A. alba* = −0.13, *P. cembra* = −0.02, *P. mugo* = −0.21), reflecting the excess of low-frequency SNP alleles, indicating population size expansion and/or purifying selection or selective sweep, whereas a positive average Tajima’s *D* value was found in *L. decidua* (1.02), indicating a decrease in population size and/or balancing selection. Fay and Wu’s normalized *H* value was negative in all species (*A. alba* = −0.13, *L. decidu* = −0.26, *P. cembra* = −0.48, *P. mugo* = −0.28), showing the excess of high-frequency-derived SNP alleles. Estimates of average Tajima’s *D* were calculated for the control genes, and estimates for *A. alba* (−0.44) and *P. mugo* (−0.42) were more than twice that of the candidate genes. Furthermore, the average Fay and Wu’s normalized *H* estimates in the candidate genes ranged from −0.13 in *A. alba* to −0.48 in *P. cembra*. In candidate genes, the average estimates of Ewens–Watterson *F* ranged from 0.61 in *P. mugo* to 0.81 in *P. cembra*. The average estimates of Kelly’s Zns were generally lower in control genes than in the candidate genes, with the exception of *P. cembra*. In the control genes, Zns ranged from 0.59 in *P. mugo* to 0.92 in *P. cembra* and varied from 0.66 in *P. mugo* to 0.85 in *L. decidua* and *P. cembra*.

The critical *P*-values of the compound test were calculated for each gene in each species by taking into account the number of individuals and its nucleotide diversity, under the SNM assumption (Data S6). The compound DHEW test was significant for 10 candidate genes (Table 3) and eight control genes (Table S4) in just *P. cembra* and *P. mugo*. No significant DHEW values were found in *A. alba* and *L. decidua* in both gene categories.

**Table 3:** List of the outliers from the standard neutral model (SNM) across the candidate genes

Species	Gene	Putative protein	*S*^a^	*P*_D_^b^	*P*_H_^b^	*P*_EW_^b^	*P*_Zns_^a^	DHEW-P*^c^	Reference
*Pinus cembra*	0_18619	Protein kinase family protein	6	0.0207	0.0257	0.8835	0.8812	0.0622	
	0_2775	spx domain-containing protein	2	0.118	0.0773	0.8062	1	0.2949	Wang et al. 2004
	0_8111	6-phosphogluconate dehydrogenase	3	0.0798	0.0331	0.9009	1	0.1807	Dal Bosco et al. 2004
	2_1528	Reduced epidermal fluorescence 4	3	0.0491	0.0829	0.7472	0.5466	0.2256	
	2_6731	E3 ubiquitin complex protein	3	0.0451	0.0169	0.746	0.5456	0.2276	Lozano-Durán et al. 2011 Bentsinka and Koornneef 2008
	CL1659Contig1	Chloride channel-like protein	4	0.0786	0.0533	0.9346	0.9552	0.1050	
	CL1661Contig1	Acetyl-CoA carboxylase 2	3	0.0453	0.0851	0.9167	0.8924	0.2272	Li-Beisson et al. 2010
*Pinus mugo*	0_13913	Exocyst subunit EXO70 family protein	4	0.1146	0.0803	0.9236	0.8508	0.0985	Synek et al. 2006
	2_8627	Carbon–sulfur lyase	10	0.1131	<0.0001	0.9846	1	0.0176	Mikkelsen et al. 2004
	2_8852	Galactokinase	8	0.0155	<0.0001	0.9919	1	0.0291	Yang et al. 2009

a The *P* value of Kelly Zns (*P*_Zn**s**_) and the number of SNPs per locus (*S*).

b Results of each test (*D* = Tajima’s *D*; *H* = Fay and Wu’s *H* and EW = Ewens–Watterson’s *F*) are presented as *P*-value.

c The critical *P*-values calculated with the compound DHEW test. (Tajima‘s D, Fay and Wu‘s H and Ewens-Watterson's F tests).

Under neutrality, the proportion of outlier loci should be the same between candidate and control genes; under non-neutrality, the proportion of candidate genes is expected to be larger than the proportion of control genes. In *P. cembra*, 4.09% the candidate genes were outliers and 2.75% the control genes were outliers; thus, there was no difference in the proportion of outliers detected between groups. In *P. mugo*, there were proportionately more outliers in the control genes (4.17%) than in the candidate genes (1.58%).

In *P. cembra*, one outlier candidate gene (locus 0_18619) encodes for a kinase family protein. Two loci (0_2775 and CL1659) encode for a transmembrane transport protein, while locus 0_8111 is involved in oxidoreductase activity. One locus (2_6731) is involved in gibberellic acid (GA) signaling. The *Arabidopsis* homologue to locus 2_1528 is involved in reduced epidermal fluorescence, while the CL1661Contig1 homologue has acetyl-CoA carboxylase activity that takes part in fatty acid biosynthetic process. In *P. mugo*, two candidate genes are involved in metabolic processes, such as carbon–sulfur lyases (locus 2_8627) and galactokinase protein (locus 2_8852), while locus 0_13913 is a member of the EXO70 gene family protein.

The distinction between candidate and control genes was made using the protein function of the Arabidopsis gene homologue with the aim to distinguish genes potentially involved in cell metabolism from unknown genes. No information were available on those latter genes; therefore, it is not possible to exclude that some control genes may be meaningful in terms of functionality. Moreover, the candidate genes were not defined according to their role in any specific adaptation to the environment, so many of these might be effectively neutral.

## Discussion

The overarching goal of this study was to compare and contrast patterns of nucleotide diversity and tests of neutrality in four conifer species of the alpine mountain region of central Europe. These results give an overview of nucleotide diversity in four coniferous species and provide a useful SNP resource that can be applied in landscape genomic studies. In spite of the result that there were no strong and consistent differences in the proportion of outliers detected between candidate and control genes, there were several putative outlier genes that may be related to environmental adaptations. A unique aspect of this study is the comparison of diversity and departure from neutrality among four tree species living in montane ecosystems.

### *Pinus cembra* showed lower diversity than the other tree species

*Pinus cembra* showed the lowest diversity among the four species, with values falling in the range of species belonging to the subgenus *Strobus*, such as *P. chiapensis* (Syring et al. 2007) and *P. albicaulis* (A. J. Eckert, A. D. Bower, K. D. Jermstad, J. L. Wegrzyn, B. J. Knaus, J. V. Syring and D. B. Neale, unpublished manuscript) and somewhat less than that found in other coniferous species (González-Martínez et al. 2006; Savolainen and Pyhäjärvi 2007). To the contrary, *P. mugo* showed the highest nucleotide diversity among the four species, with values similar to other species belonging to the subgenus *Pinus* (Grivet et al. 2009, 2011; Ma et al. 2006; Shiraishi and Shiraishi 2011; Wachowiak et al. 2009; Eveno et al. 2008*)*.

The contrasting patterns of nucleotide diversity of the two pines, growing in similar altitudinal ranges (1200–2300 m for *P. cembra* and 1000–2000 m for *P. mugo*), call for an interpretation. This result may be linked to the different demographic histories of the two pines, because *P. cembra* is characterized by two distinct postglacial refugia in the Carpathians and in the Alps (Höhn et al. 2009), whereas in the Pliocene, the large range of *P. mugo* was separated into several refugia that are poorly known ([Bibr b64][Bibr b35]). Moreover, *P. cembra*, like *P. albicaulis* Engelm., has bird-dispersed seeds (Tomback 2005), which may lead to higher levels of inbreeding (Rogers et al. 1999). Low genetic diversity within *P. cembra* populations in the northern Alps may be due to genetic drift by restricted gene flow (Gugerli et al. 2009). The nonpine species of this study, *A. alba* and *L. decidua*, had fairly high estimates of nucleotide diversity compared to two other pines, *P. sylvestris* (Pyhäjärvi et al. 2007; Palmé et al. 2008) and *P. luchuensis* (Shiraishi and Shiraishi 2011).

### *Pinus cembra* shows proportionally more outlier loci

The compound DHEW test detected the presence of outlier loci in only *P. cembra* and *P. mugo*, although the single-locus tests revealed the possible presence of outlier loci in *A. alba* and *L. decidua* as well. These results suggest that selection may have acted more in the two pines than in the other two species, although this interpretation could be confounded by the fact that proportionally more highly conserved genes were tested in *A. alba* and *L. decidua* or that *P. cembra* had the lowest average diversity.

The presence of several genes, especially in *P. mugo*, showing higher nucleotide diversity at synonymous sites compared to the other site categories is an indication of purifying selection (e.g., Palmé et al. 2009), in accordance with the expectation that in coding regions, most mutations are probably disadvantageous. Moreover, divergence at synonymous sites was three times the value at nonsynonymous sites and up to eight genes per species displayed *K*_a_/*K*_s_ ratios greater than one, which may indicate the presence of positive selection (Palmé et al. 2008). In the candidate genes, the negative average estimate of Tajima’s *D* found in *A. alba* and in the two pines may indicate the presence of recent demographic events, such as population size expansion or purifying selection or selective sweeps. Moreover, the positive value of Tajima’s D in *L. decidua* may indicate a decrease in population size and/or balancing selection.

Several loci deviating from neutrality were found in both control and candidate gene sets in *P. cembra*. Among candidate outlier loci, locus 2_6731 is the most interesting as its homologue encodes for the E3 ubiquitin complex protein, an F-box protein that is involved in GA signaling. Ubiquitination controls most of the hormonal responses in plants and is one of the dominant plant regulatory mechanisms (reviewed in Dreher and Callis 2007; Santner and Estelle 2009). Plant DNA viruses (Geminiviruses) may interfere with several responses regulated by the ubiquitin E3 ligases, making the plant more susceptible to virus infection (Ascencio-Ibáñez et al. 2008; Lozano-Durán et al. 2011). Moreover, GA modulates plant growth and development throughout the whole lifecycle of the plant (Sun 2010). Additionally, two outliers encoded for proteins related with membrane transporters (loci 0_2775, CL1659Contig1). In particular, the *Arabidopsis* homologue of locus 0_2775 is involved in cellular uptake of inorganic phosphate in the root xylem (Wang et al. 2004). In the same species, one outlier (locus CL1661Contig1) encodes for acetyl-CoA carboxylase, the enzyme that catalyzes the first committed step in fatty acid synthesis (Konishi et al. 1996; Li-Beisson et al. 2010). Acyl lipids constitute the membrane between cell and organelles. These genes may be important for tree fitness, because organelle proteins change in abundance during stress, as an immediate response to abiotic stress (Taylor et al. 2009).

Several outliers were also found in both control and candidate gene sets in *P. mugo*. Among the candidate gene outliers, locus 0_13913 encodes for a member of EXO70 family protein, which is involved in exocytosis. One member of this gene family (AtEXO70A1) was found to be crucial for polar growth and plant development (Synek et al. 2006). Locus 2_8627 has a catalytic activity for the carbon–sulfur lyase that is involved in glucosinolate biosynthesis (Mikkelsen et al. 2004). Glucosinolates are amino acid-derived natural plant products in *Arabidopsis*, implicated in plant defense (Halkier and Gershenzon 2006). Locus 2_8852 encodes for a galactokinase that is involved in the synthesis of d-galacturonic acid (d-GalA) polysaccharides (Yang et al. 2009).

To compare our results with other coniferous species and between species in this study, it is important to consider our results in the context of possible biases owing to (i) sequence conservation that determined the number of loci successfully resequenced, (ii) number of trees sampled, (iii) species range covered by the sampling and (iv) the effect of demography that differs from the SNM assumptions. The much lower success in the resequencing in *A. alba* and *L. decidua* than in the two pines was a direct effect of sequence conservation with *P. taeda* from which primers were designed. The unbalanced number of sequences per tree may have affected the estimates of nucleotide diversity, especially in *L. decidua*, which had the lowest average number of sequences per gene (*n* = 3 ± 1). In the same species, the low number of reads may have biased outlier detection; nevertheless, the sample number was used in the estimation of the neutrality tests.

Furthermore, the small number of trees sampled, the partial coverage of species ranges, and a nonuniform sample distribution according to species demographic history could all affect estimates of diversity (Städler et al. 2009). *Abies alba* was sampled mainly in the central-west of Europe; this might bias the estimation of nucleotide diversity, because there is a clear separation into two maternal lineages in *A. alba* (Liepelt et al. 2009). In *P. cembra*, more trees were sampled in the Alps than in the Carpathian Mountains. These two areas belonged to two different lineages, with the Carpathian populations being more polymorphic than the populations in the Alps (Belokon et al. 2005; Höhn et al. 2009). For *P. mugo*, the sampling covered the species range, including the area in which the different varieties (*P. mugo s.s.* and *P. uncinata*) overlap. Moreover, for the identification of the outlier loci, the estimation of the *P*-value in the neutrality tests did not take into account species demography. It should be noted, however, that the compound test is fairly robust to demographic deviations from the SNM (Zeng et al. 2006, 2007). The bias in sequence conservation may also have affected the identification of the outliers from SNM in the studied species, because the percentage of outliers per gene set per species ranged from 4.17% to 1.58% in the two pines, while no outliers were identified in *A. alba* and *L. decidua*.

## Conclusions

This study is a first step toward trying to understand the molecular basis of adaptation, both lineage-wide and locally, for these alpine conifers. Patterns of nucleotide diversity showed that the two pines sharing the same high altitudinal habitat had contrasting levels of diversity, while the nonpine species had intermediate values. The low nucleotide diversity and the abundance of outlier loci found in *P. cembra* compared to the two nonpine species and to the other pine *P. mugo* may suggest that *P. cembra* may have gone through different demographic events that may have changed the original population size. Therefore, *P. cembra* might be more susceptible to changing climate, not having sufficient diversity to adapt to changing environment.

This research is an exploratory study on genetic diversity in four forest species that provides a new set of genetic markers. Among the present methods for the SNP discovery in nonmodel species, the candidate gene approach (Sanger sequencing) is widely used and generally requires primers that are specific to the target gene (Garvin et al. 2010). In this research, a set of candidate genes, first developed in *P. taeda*, was successfully transferred to other coniferous species for SNPs discovery. For example, the candidate gene approach was applied to investigate plant adaptation to drought in *P. taeda* (González-Martínez et al. 2006). A more recent and powerful approach, although not available at the time of this study, is RNA-Seq technology (Wang et al. 2009). A recent example of RNA-Seq technology applied to a conifer is that in *P. contorta* (Parchman et al. 2010).

This study is a first step in developing a polymorphism resource for four important nonmodel species of European Alpine forests. Future studies will focus on SNP genotyping across a large geographic area, with the goal of understanding the relationship between tree genotype and environmental factors, such as altitude, temperature, and water availability.

## Data Archiving Statement

Raw data sequences are currently available in the following database at the University of California: http://loblolly.ucdavis.edu/bipod/ftp/Sanger_resequencing/. In this database, the files are separated according to species and then locus. The studied species are coded in this way: *Abies alba* (Abal), *Larix decidua* (Lade), *Pinus cembra* (Pice), and *Pinus mugo* (Pimg). The samples are coded with the Acesap project name. The sequence data underlying the main results of the study were submitted to the GenBank website (http://www.ncbi.nlm.nih.gov/genbank/). These sequences were accepted by GenBank, and their accession number goes from JQ440374 to JQ445205. Sequences shorter than 200 bp were submitted to EMBL website (https://www.ebi.ac.uk/Databases/). The accession number for those sequences goes from HE663538 to HE663608 and from HE681087 to HE681096.

## Supporting Information

**Data S1.** Primer sequences.

**Data S2.** All studied genes and their blast.

**Data S3.** DnaSAM results for each gene.

**Data S4.** Annotation input for each gene.

**Data S5.** PolyDNdS output and gestimator output.

**Data S6.** List of outliers for the compoundneutrality test (DHEW-*P**).

**Figure S1.** Flow-chart of the data generation and screening.

**Figure S2.** Distribution of nucleotide diversity (θ_w_, θ_π_) in control gene forthe different species (A) and distribution of divergence from loblolly pine across several site categories (B).

**Figure S3.** Distribution of nucleotide diversity (θ_w_, θ_π_) in candidate genes (A) and control ones (B) across several site types in the four species.

**Figure S4.** Frequency plots of the number of SNP per gene in the candidate genes for each species.

**Table S1.** List of the species samples with their geographical location.

**Table S2.** Estimates of nucleotide diversity in the control genes for several site types.

**Table S3.** Estimates of the neutrality tests per gene set (candidate versus control) for each species.

**Table S4.** List of the outliers from the standard neutral model (SNM) across the control genes.
