# Supplementary figures and images for: Contrasting patterns of nucleotide diversity for four conifers of Alpine European forests
Source: Evol Appl. 2012 Nov;5(7):762–75. doi: 10.1111/j.1752-4571.2012.00256.x (PMC3492901; doi:10.1111/j.1752-4571.2012.00256.x)

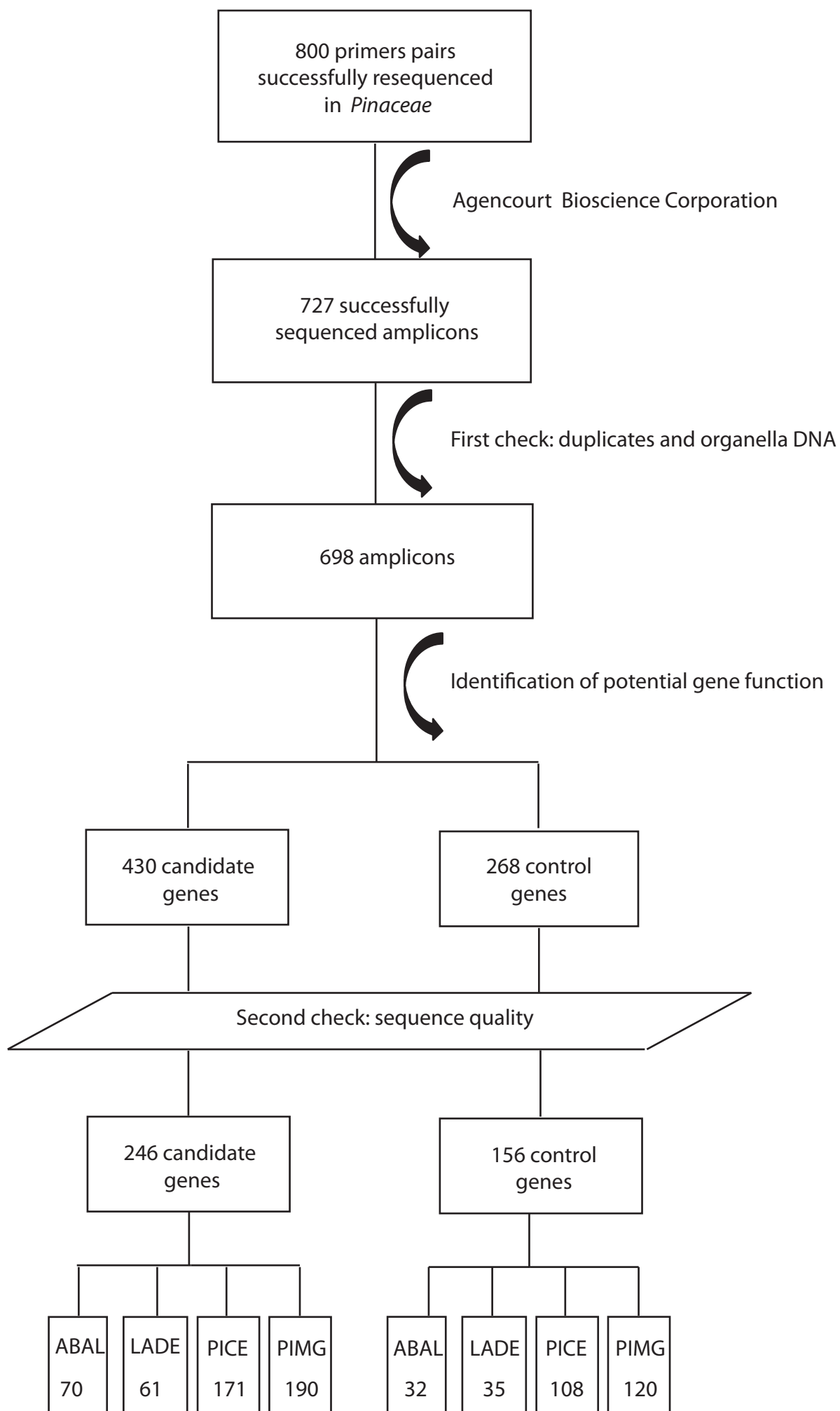

Supplement: Supplementary file 7 [file eva0005-0762-SD1.pdf]

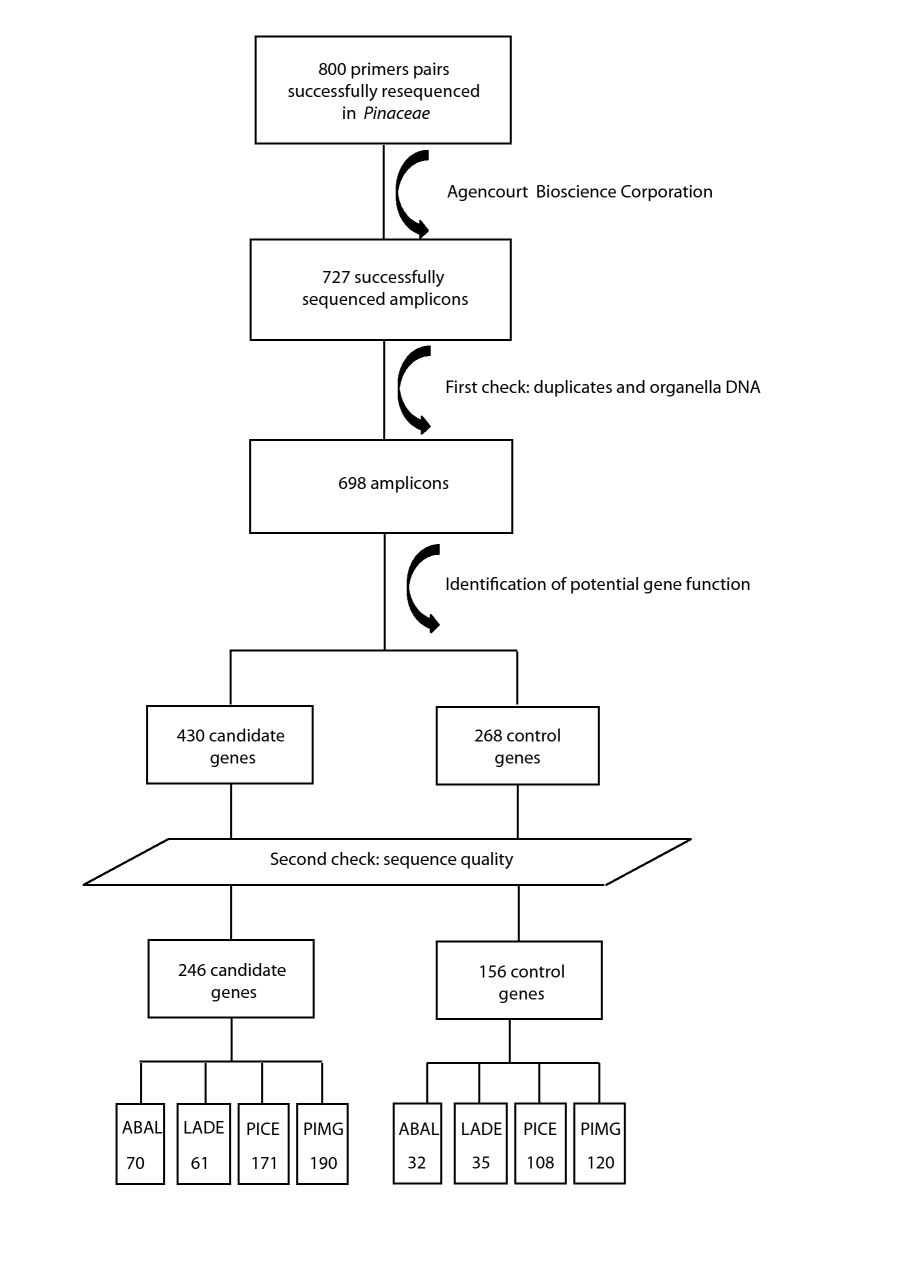

Supplement: Supplementary file 9 [file eva0005-0762-SD14.png]

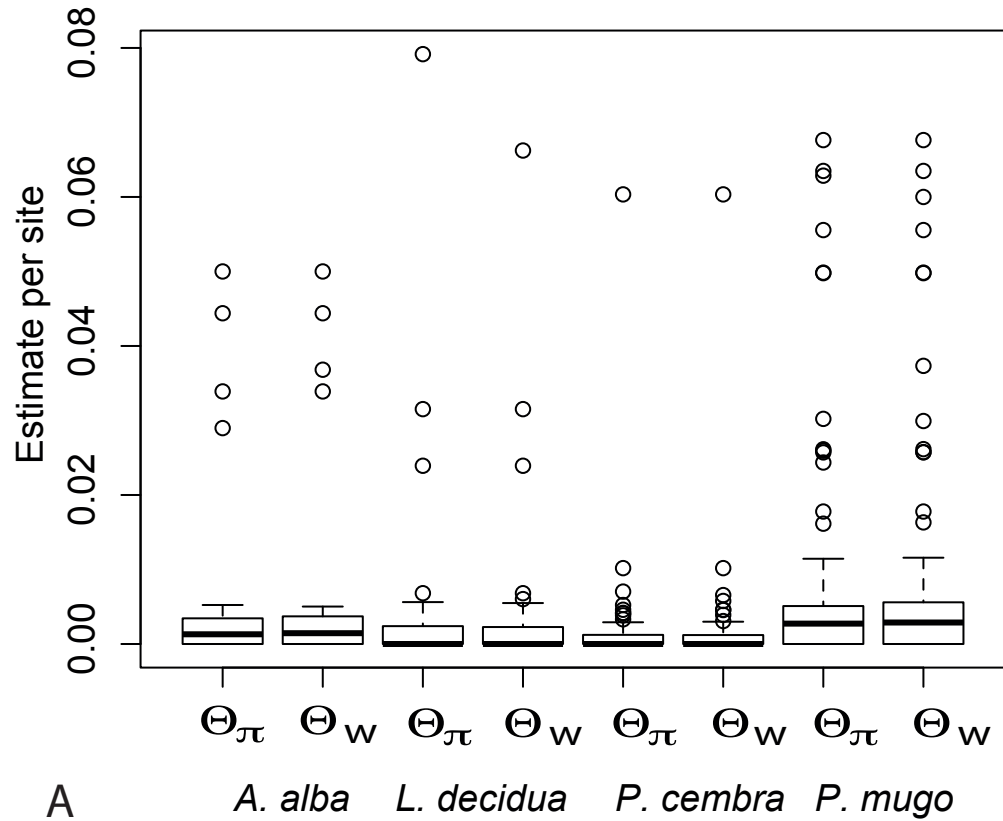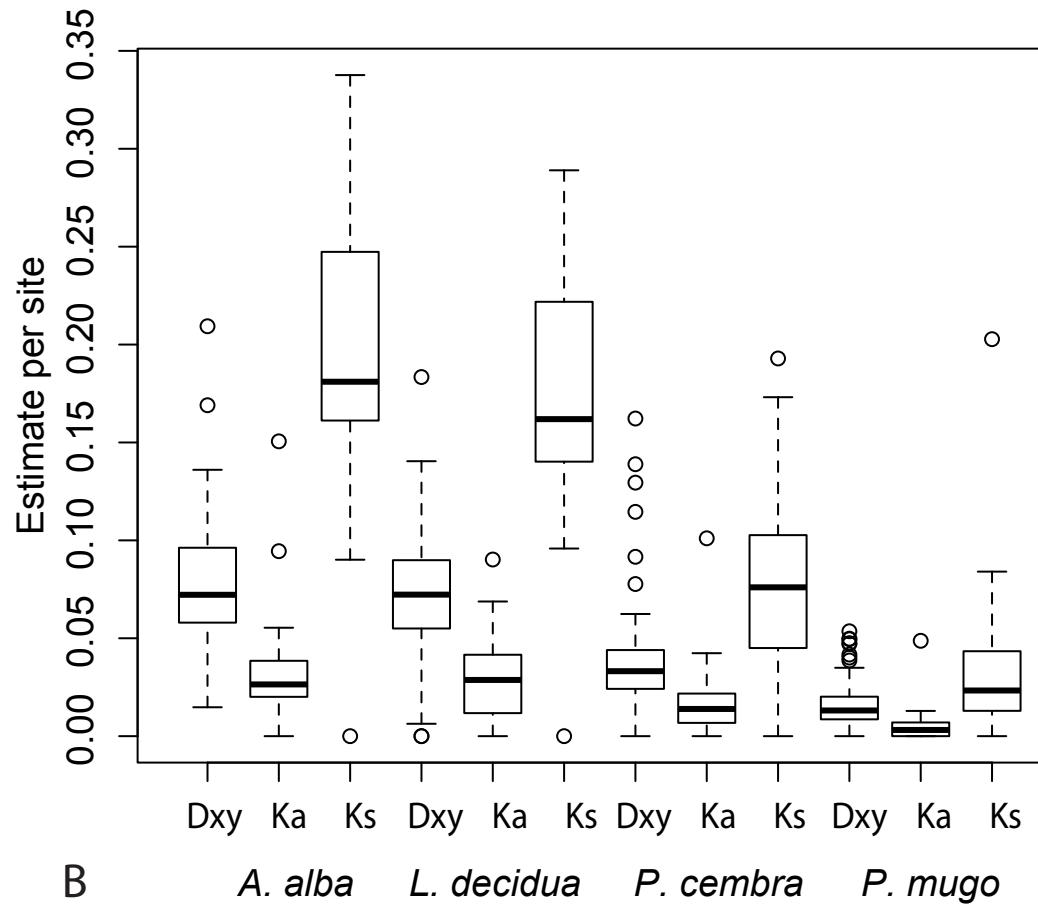

Supplement: Supplementary file 11 [file eva0005-0762-SD2.pdf]

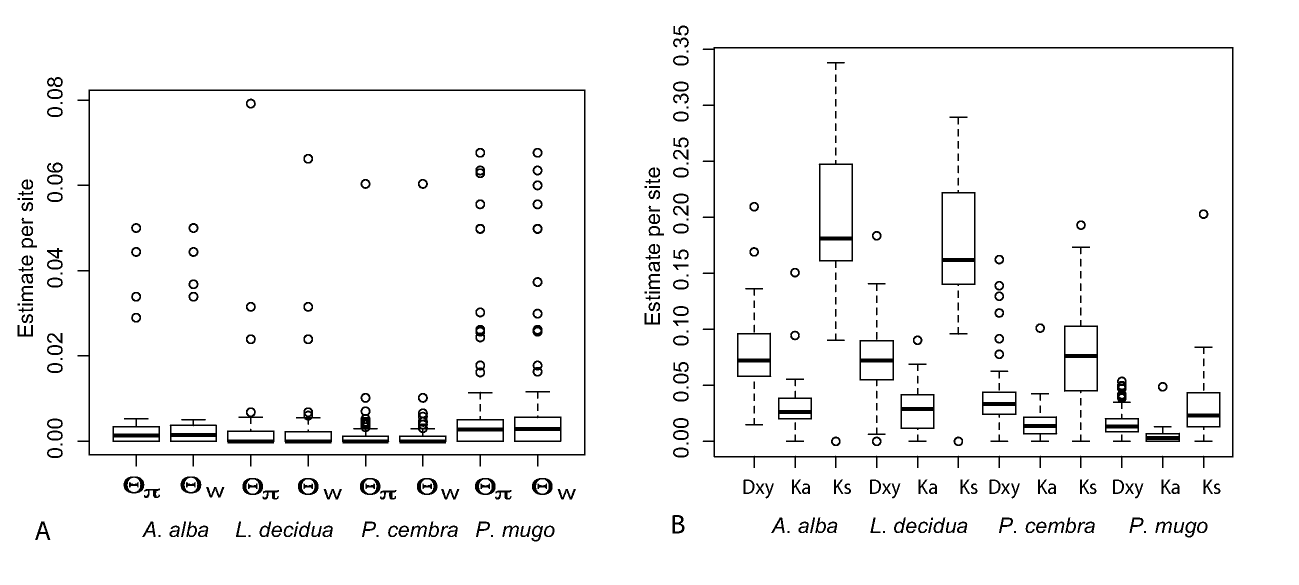

Supplement: Supplementary file 12 [file eva0005-0762-SD15.png]

*Abies alba*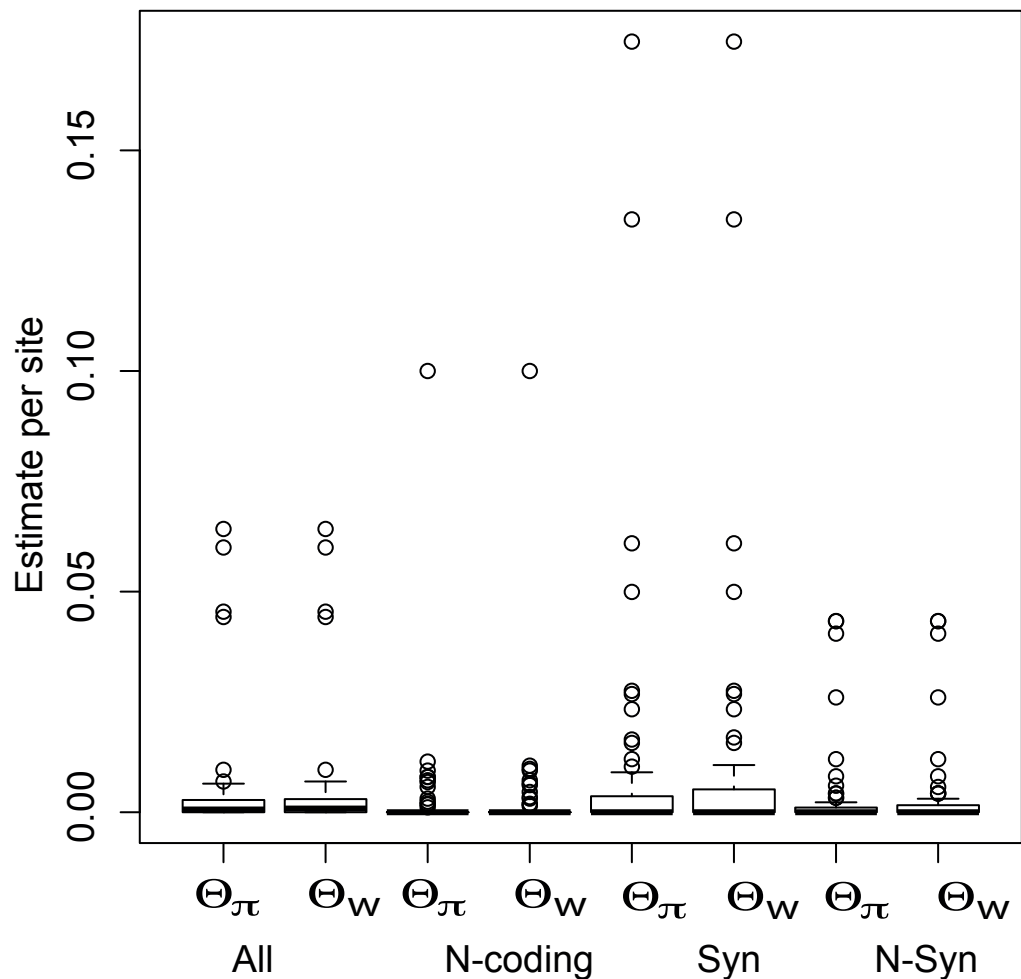*Larix decidua*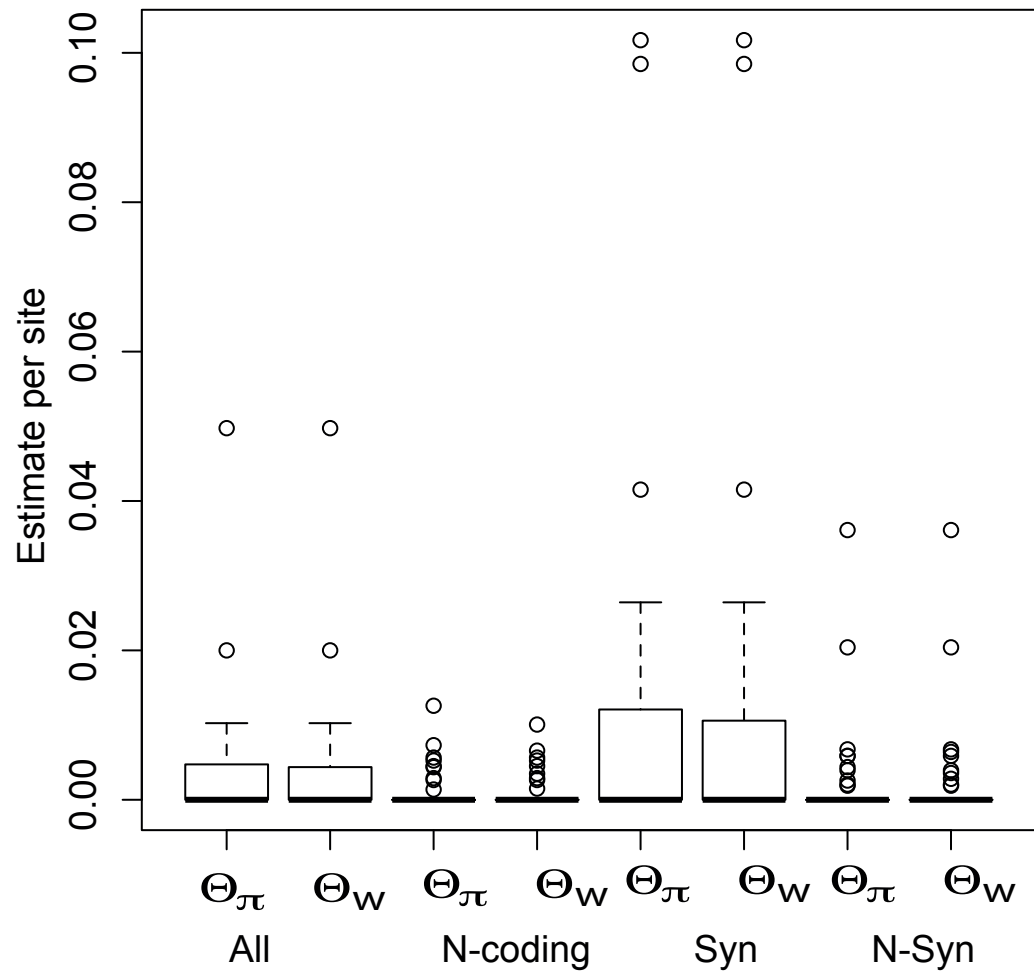*Pinus cembra*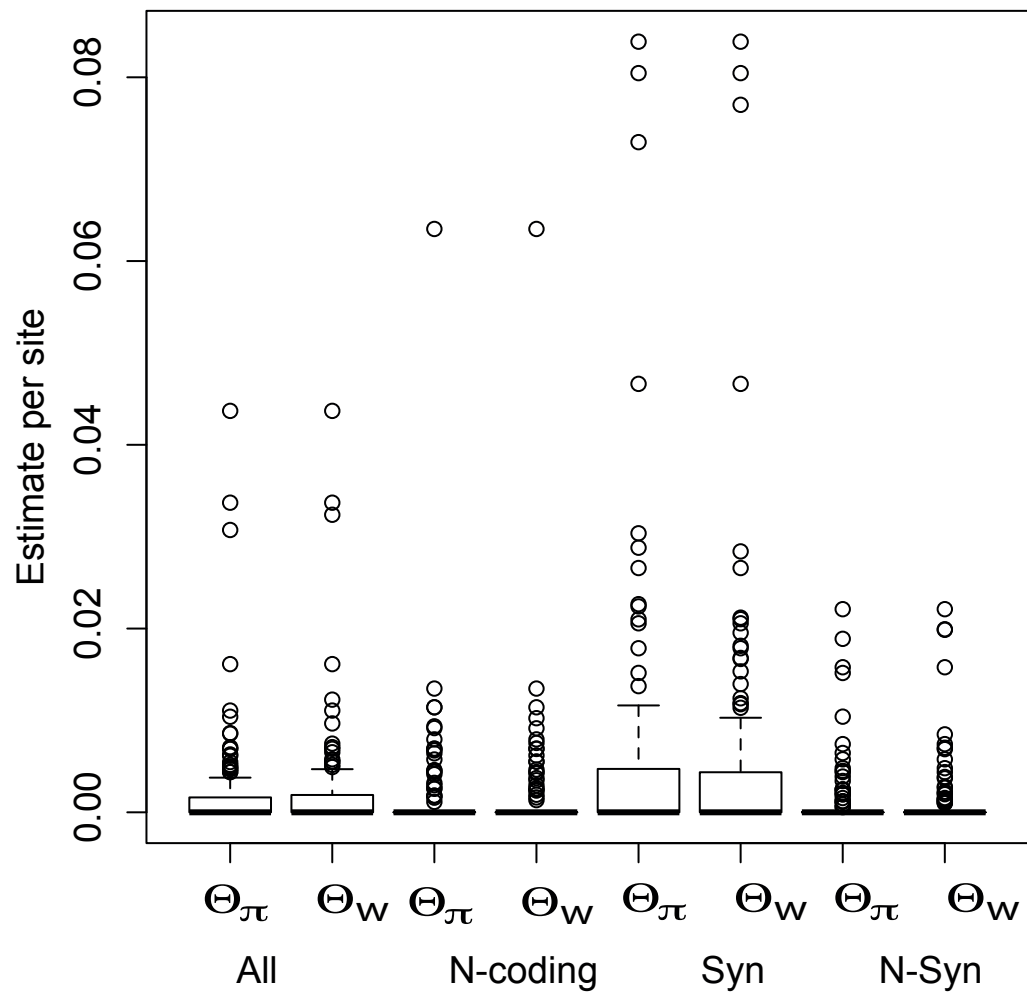*Pinus mugo*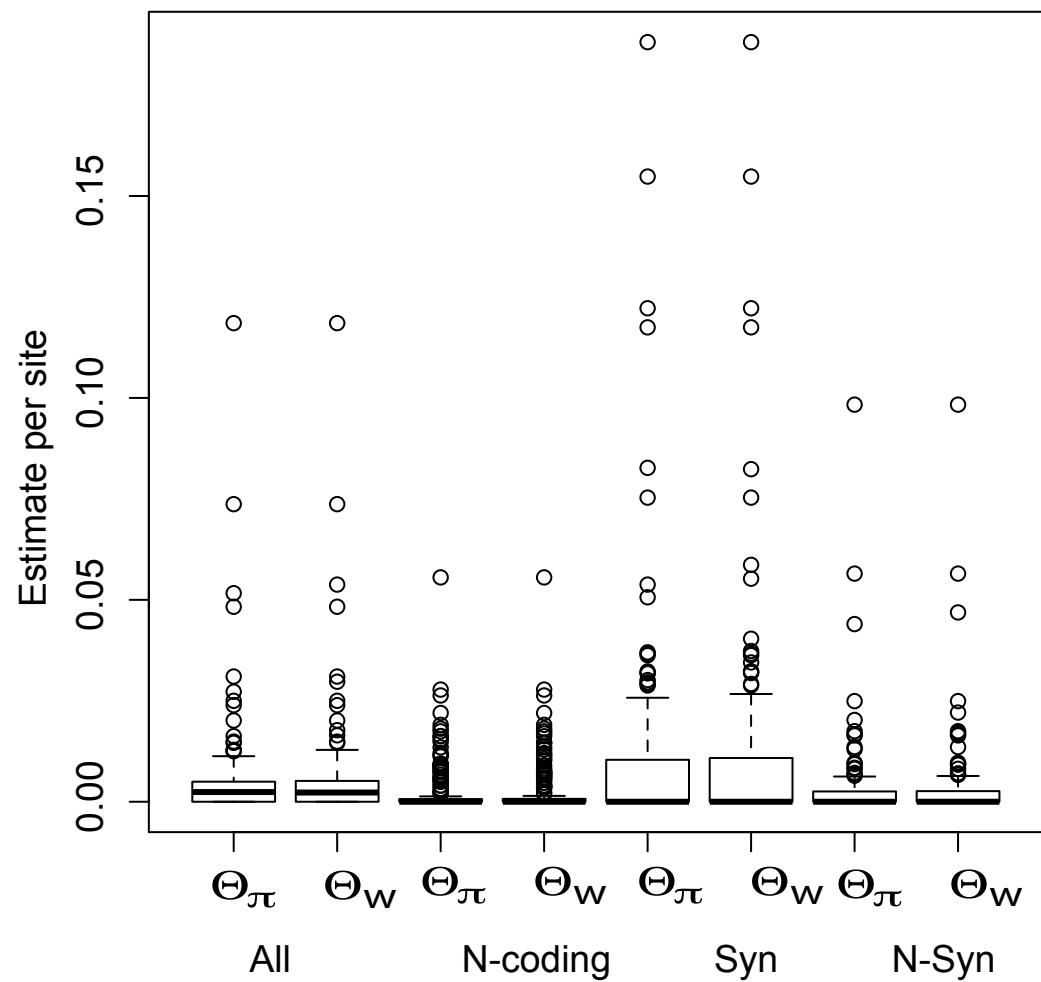

Supplement: Supplementary file 14 [file eva0005-0762-SD3.pdf]

*Abies alba*

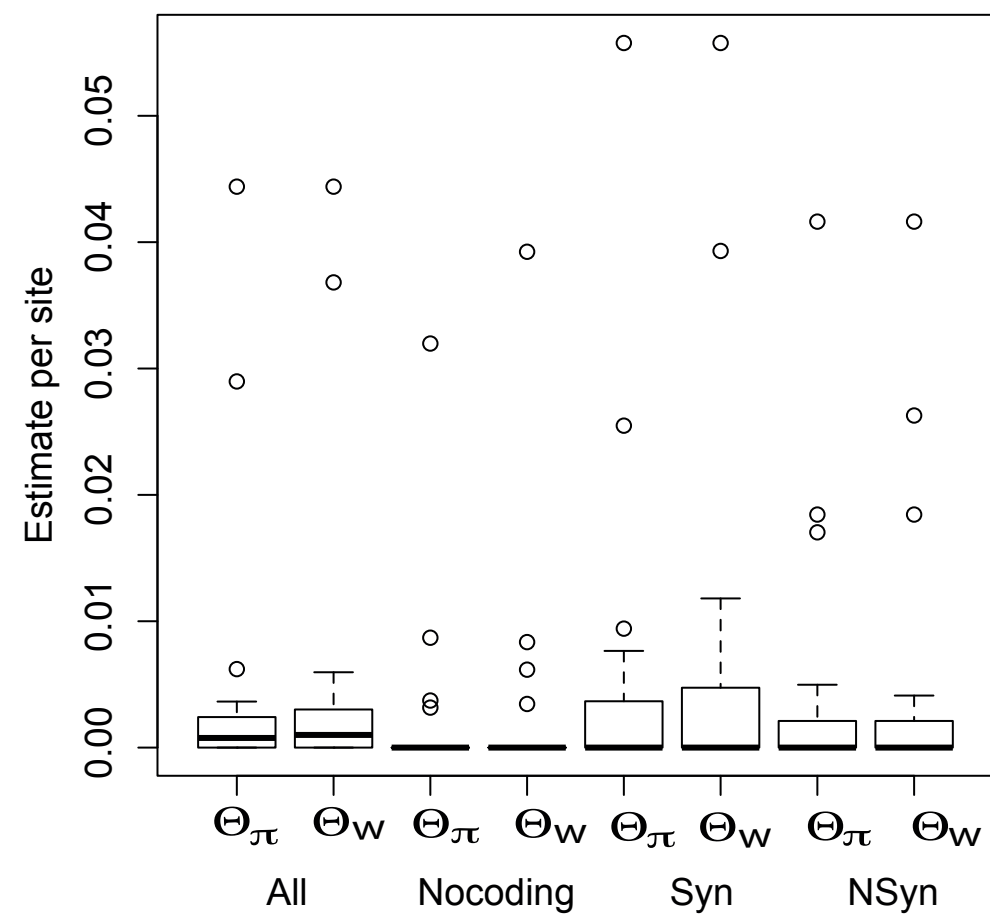

*Larix decidua*

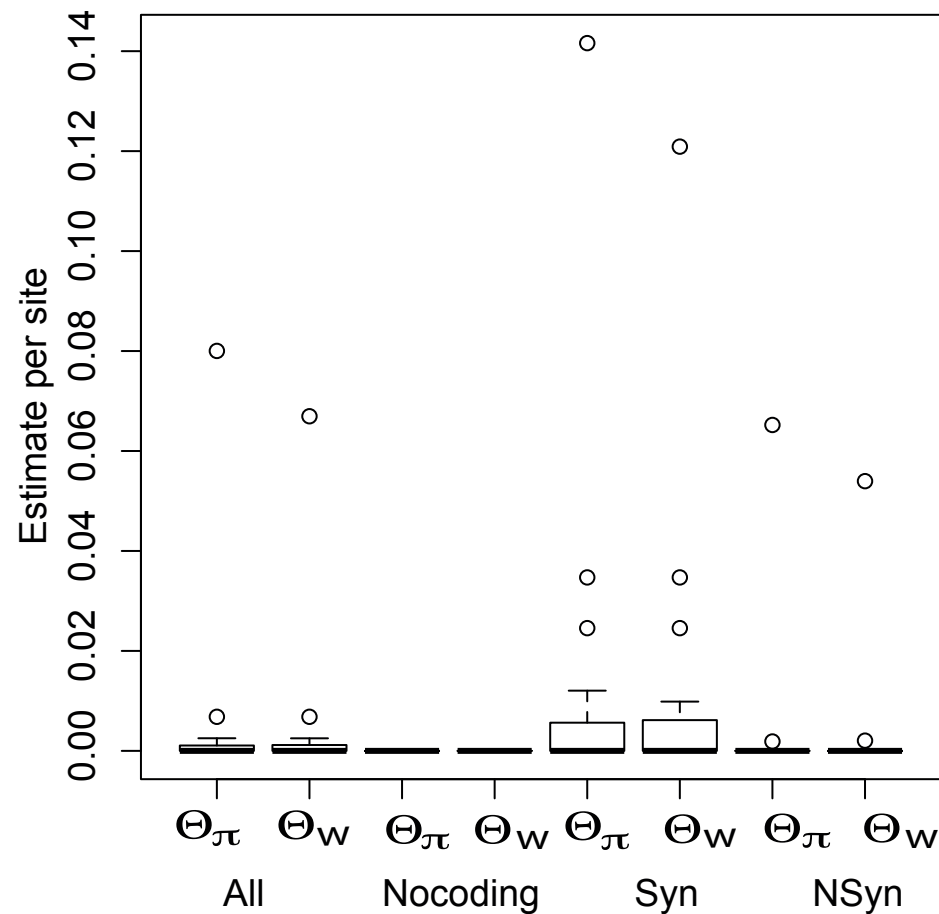

*Pinus cembra*

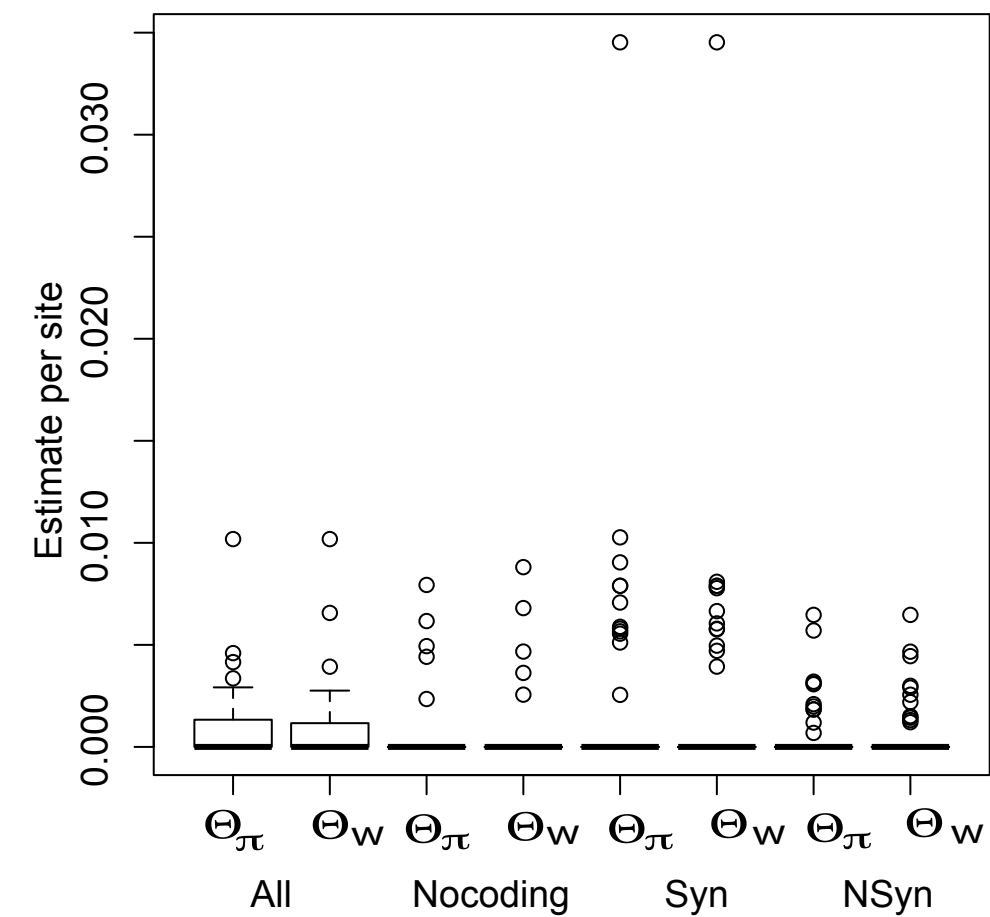

*Pinus mugo*

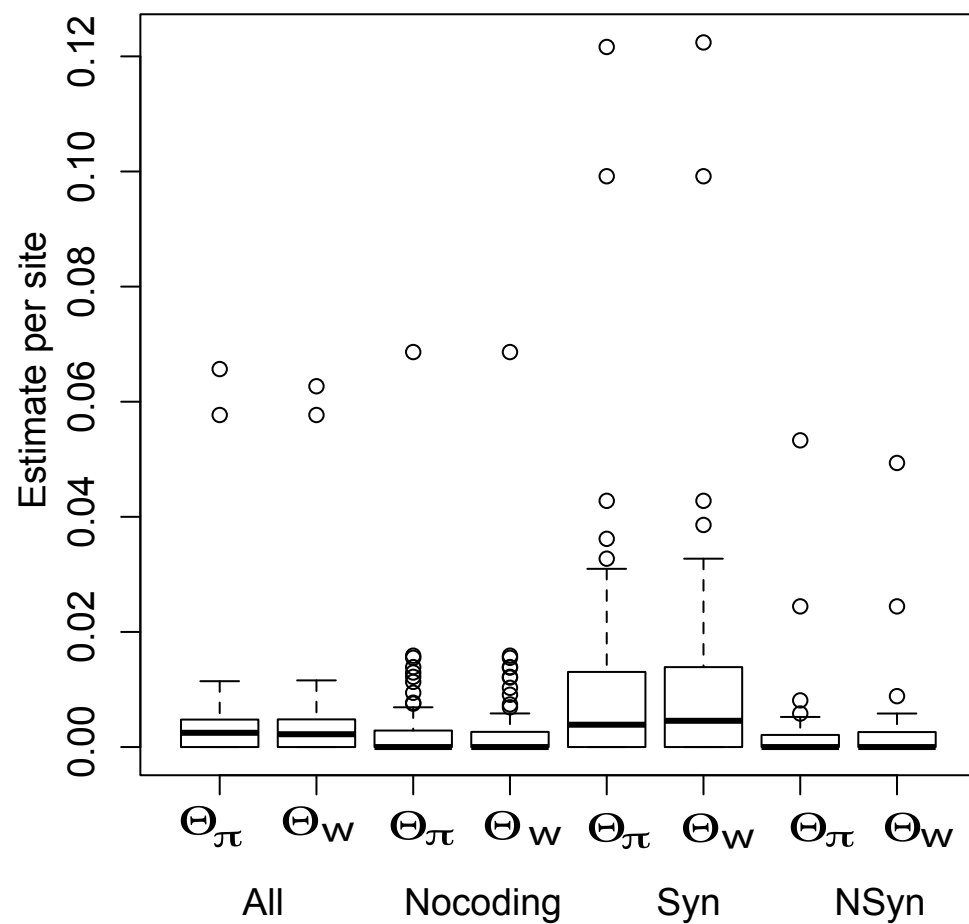

Supplement: Supplementary file 15 [file eva0005-0762-SD4.pdf]

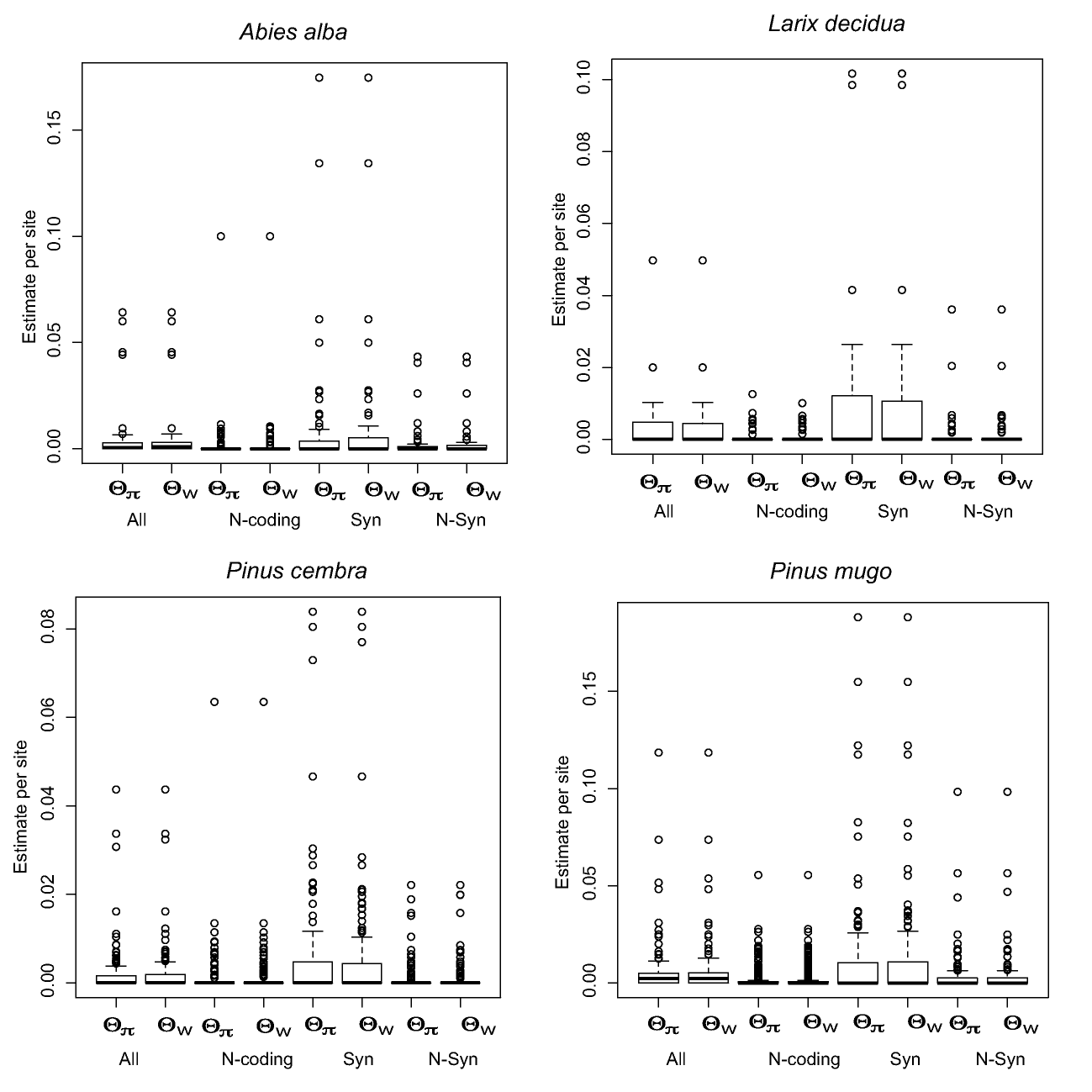

Supplement: Supplementary file 16 [file eva0005-0762-SD16.png]

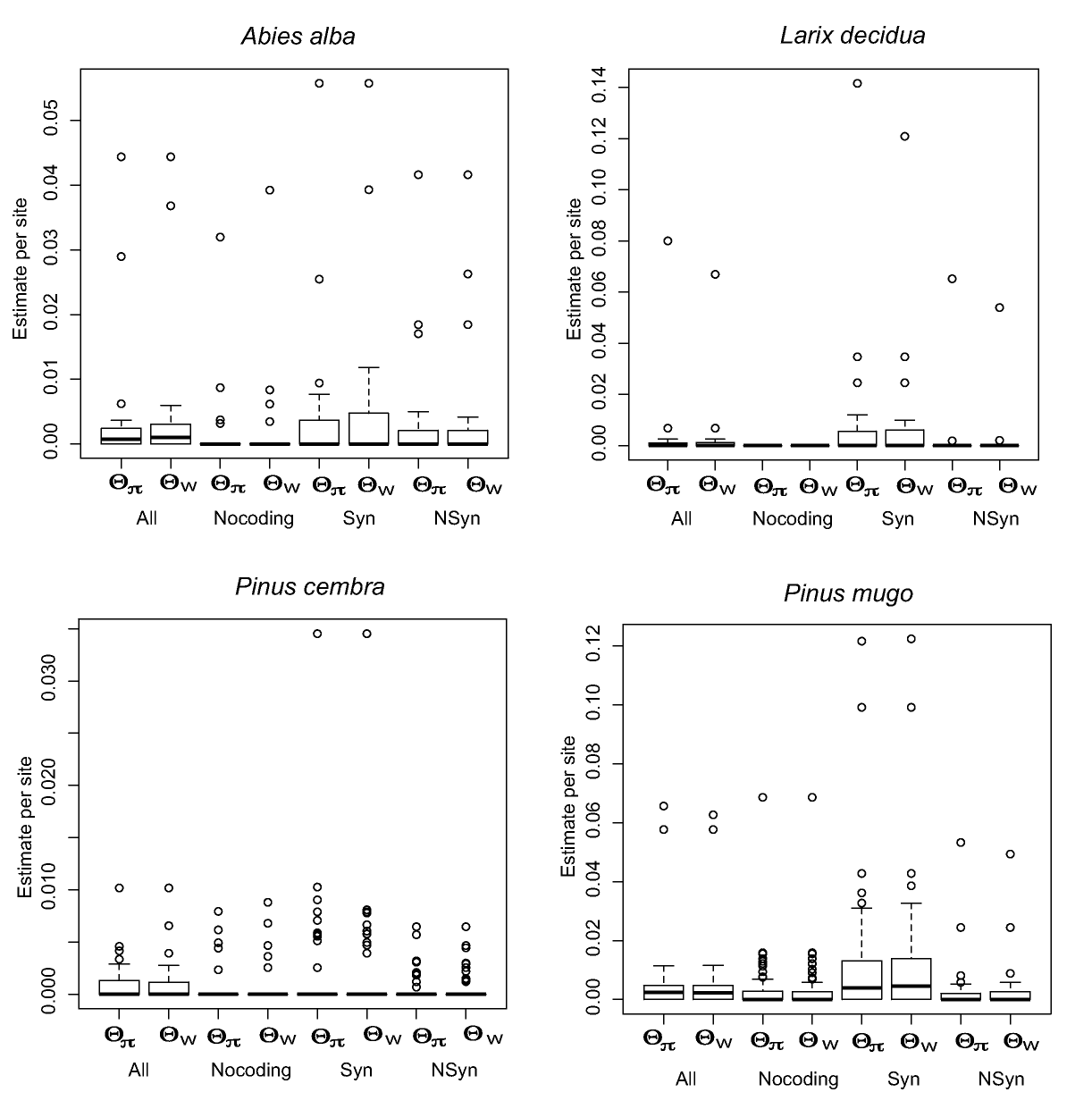

Supplement: Supplementary file 17 [file eva0005-0762-SD17.png]

*Abies alba*

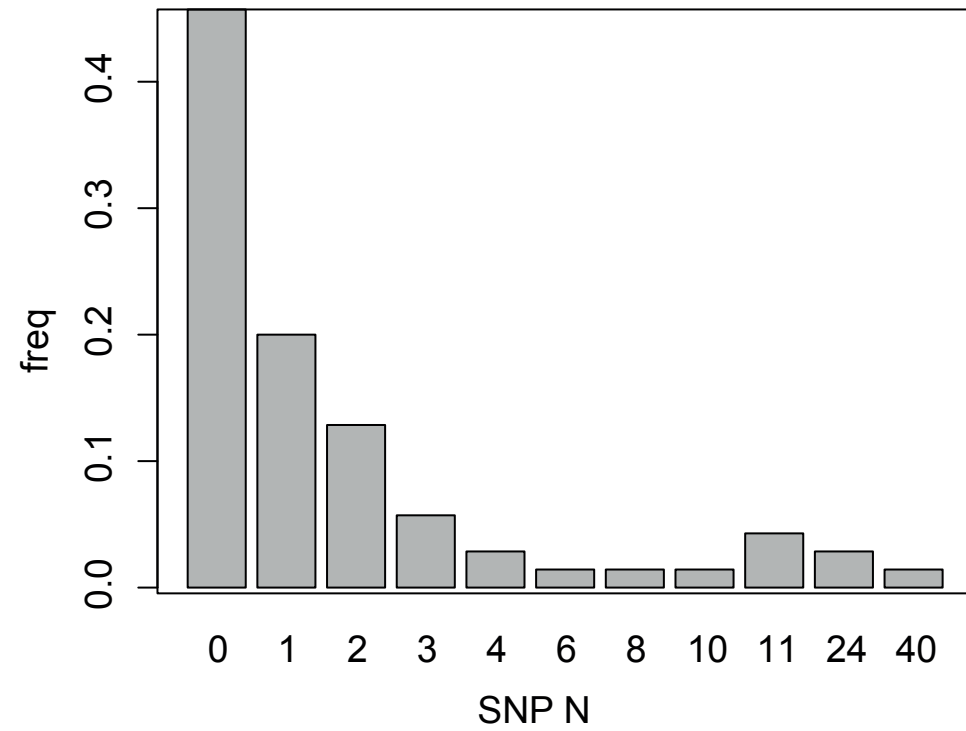

*Larix decidua*

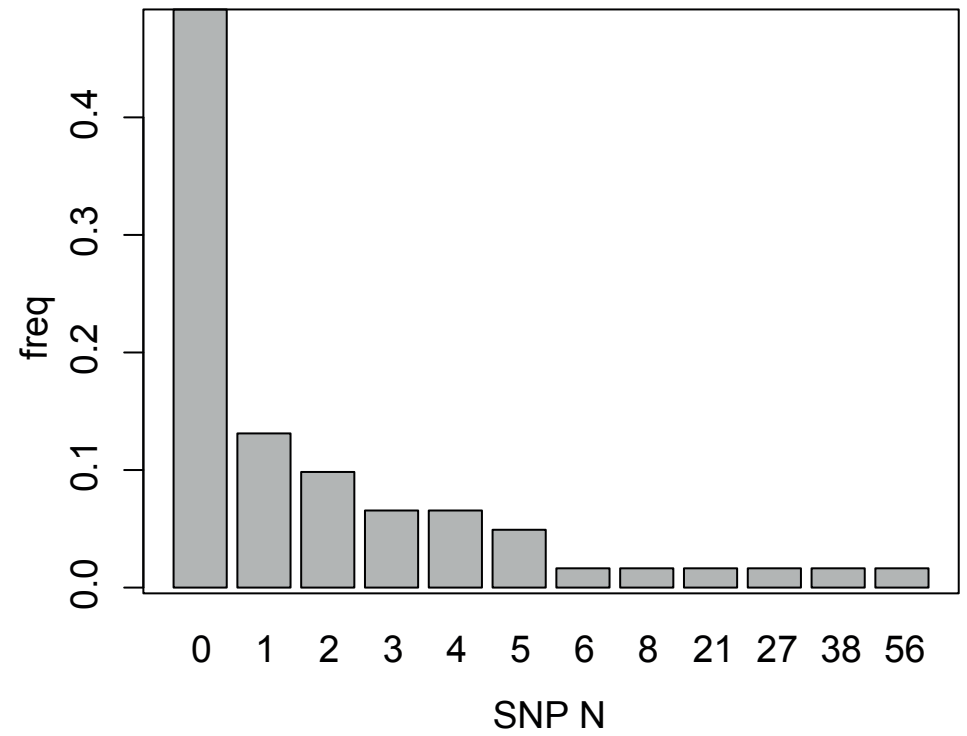

*Pinus cembra*

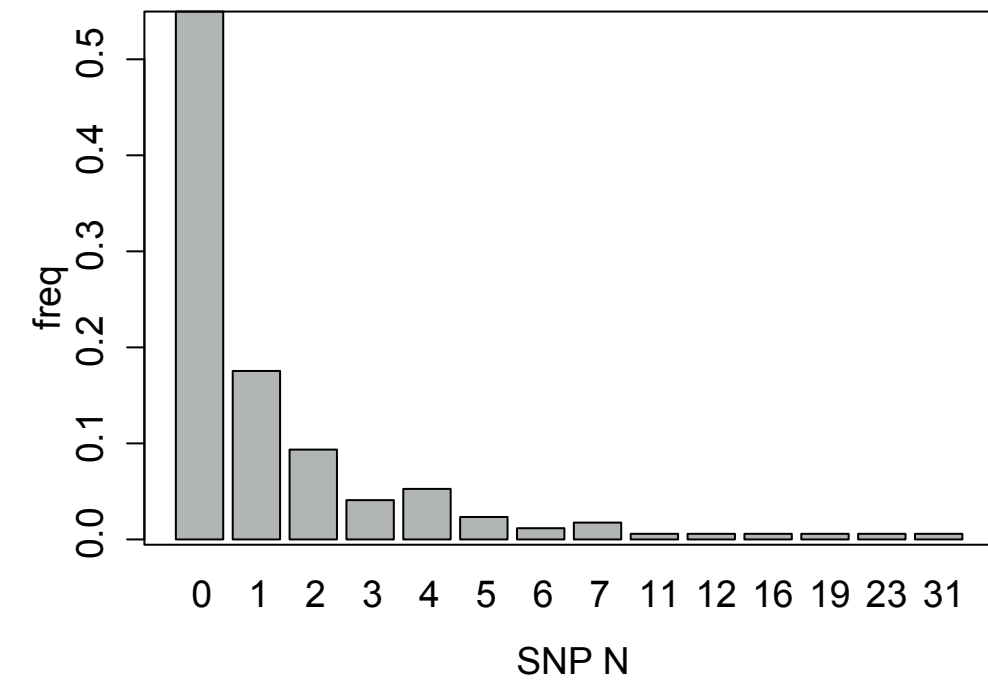

*Pinus mugo*

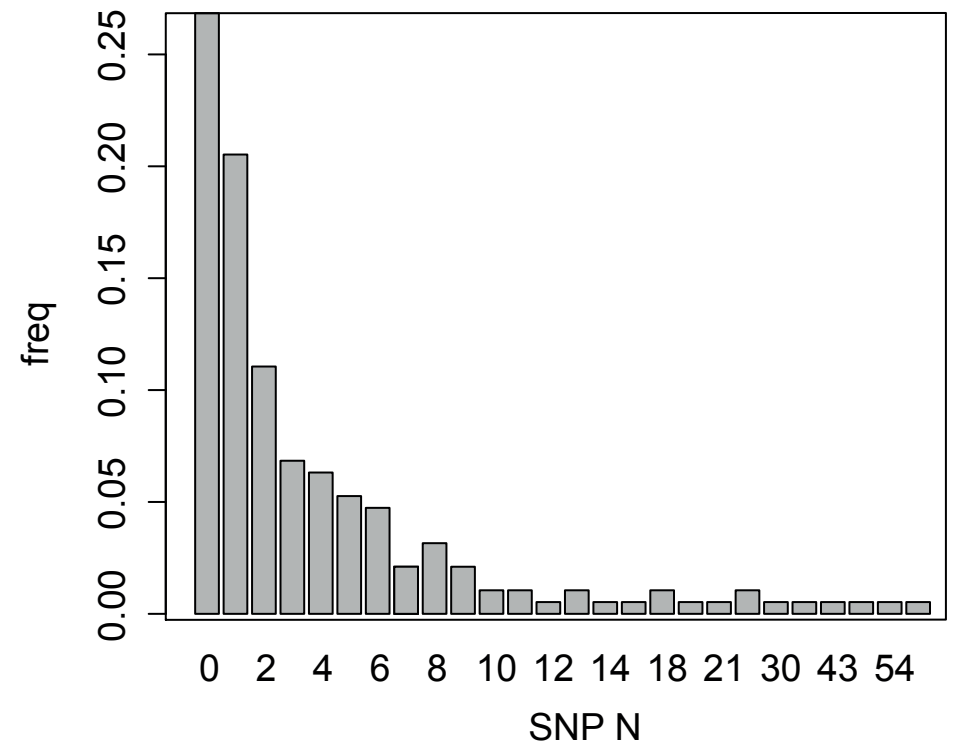

Supplement: Supplementary file 20 [file eva0005-0762-SD5.pdf]

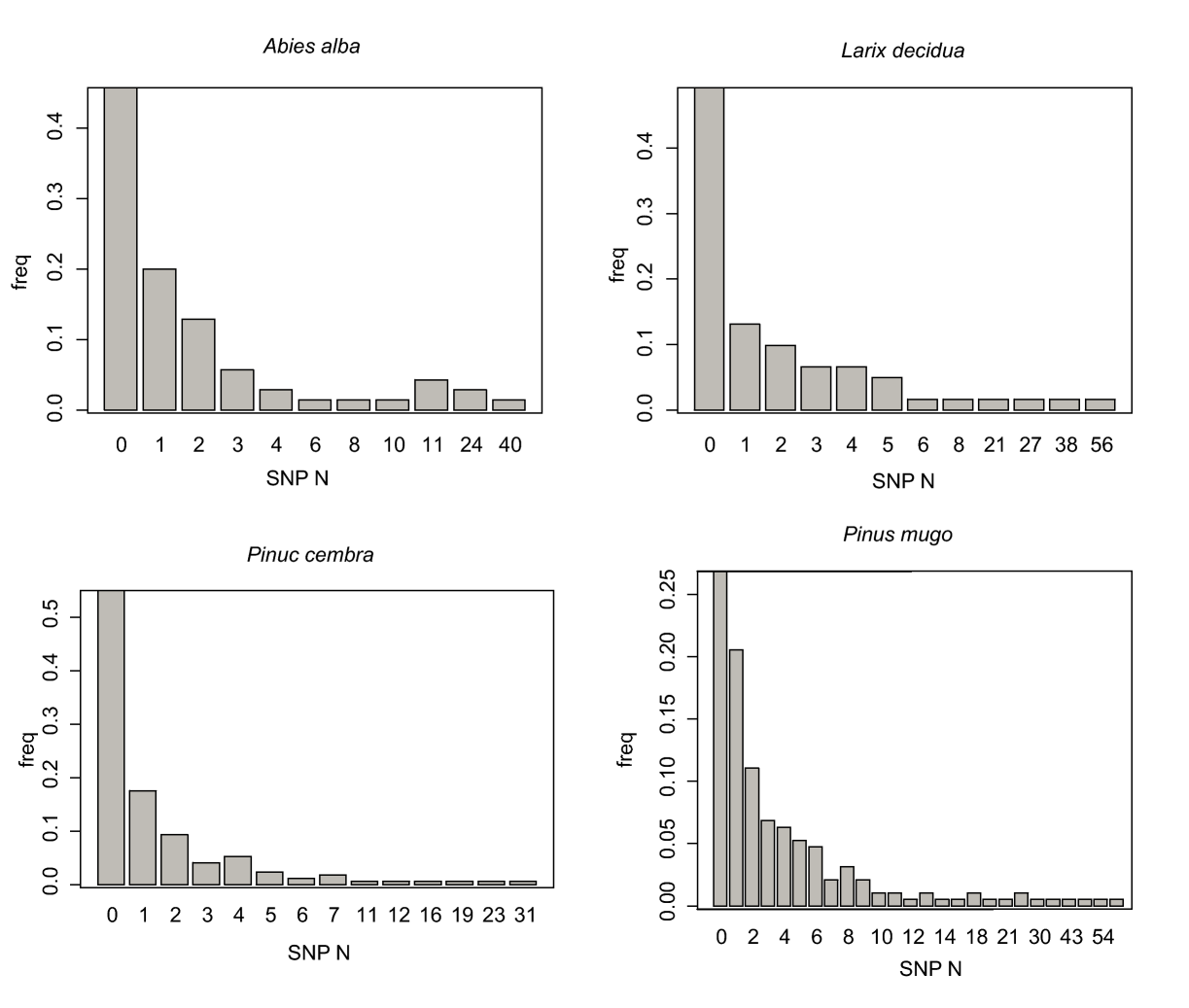

Supplement: Supplementary file 21 [file eva0005-0762-SD18.png]
