# Supplementary material for: Contrasting patterns of nucleotide diversity for four conifers of Alpine European forests
Source: Evol Appl. 2012 Nov;5(7):762–75. doi: 10.1111/j.1752-4571.2012.00256.x (PMC3492901; doi:10.1111/j.1752-4571.2012.00256.x)
Supplement: Supplementary file 8 [file eva0005-0762-SD12.doc]

**Supplementary figures**

Fig. S1: Flow-chart of the data generation and screening. Species names are indicated by the following codes: ABAL (*A. alba*). LADE (*L. decidua*), PICE (*P. cembra*), PIMG (*P. mugo*).

Fig. S2: Distribution of nucleotide diversity (θ_w_, θ_π_) in control gene for the different species (A) and distribution of divergence from loblolly pine across several site categories (B). D_xy_, divergence at all sites; K_a_, divergence at non-synonymous sites; K_s_, divergence at synonymous sites.

Fig. S3: Distribution of nucleotide diversity (θ_w_, θ_π_) in candidate genes (A) and control ones (B) across several site types in the four species. All, all sites; N-coding, non coding sites; N-Syn, non synonymous sites; Syn, synonymous sites.

Fig. S4: Frequency plots of the number of SNP per gene in the candidate genes for each species.
