# Supplementary material for: Contrasting patterns of nucleotide diversity for four conifers of Alpine European forests
Source: Evol Appl. 2012 Nov;5(7):762–75. doi: 10.1111/j.1752-4571.2012.00256.x (PMC3492901; doi:10.1111/j.1752-4571.2012.00256.x)
Supplement: Supplementary file 23 [file eva0005-0762-SD13.doc]

**Supplementary tables**

Table S1: List of the species samples with their geographical location.

| **Species** | **Sample ID** | **Locality ID** | **Latitude** | **Longitude** | **Country** |
| --- | --- | --- | --- | --- | --- |
| *Abies alba* | A1 | Massif Central | N 45.78 | E 2.96 | France |
|  | A2 | Mont Ventoux_532 | N 44.18 | E 5.28 | France |
|  | A3 | Oriental Pyrenees | N 42.43 | E 1.37 | Spain |
|  | A4 | Mont Ventoux_542 | N 44.18 | E 5.28 | France |
|  | A5 | French Pyrenees | N 42.78 | E 1.19 | France |
|  | A6 | Central Pyrenees | N 42.78 | E 0.31 | Spain |
|  | A7 | Black Forest_R10 | N 48.53 | E 8.03 | Germany |
|  | A8 | South Germany_F10 | N 47.71 | E 11.63 | Germany |
|  | A9 | South Germany_J1 | N 47.62 | E 10.90 | Germany |
|  | A10 | Hinterhornbach | N 47.37 | E 10.50 | Austria |
|  | A11 | Gosau-Mittertal | N 47.57 | E 13.54 | Austria |
|  | A12 | Corsica | N 42.16 | E 9.10 | France |
|  |  |  |  |  |  |
| *Larix decidua* | L1 | Tartu Parish | N 58.41 | E 26.79 | Estonia |
|  | L2 | Coceana | N 44.82 | E 25.95 | Romania |
|  | L3 | Austria_101_22 | N 47.40 | E 14.86 | Austria |
|  | L4 | Austria_101_06 | N 47.40 | E 14.86 | Austria |
|  | L5 | Austria_131 | N 47.30 | E 15.21 | Austria |
|  | L6-L12 | French plates | N 44.50 | E 6.00 | No reference |
|  |  |  |  |  |  |
| *Pinus mugo* | PM1 | Rila Mountain | N 42.10 | E 23.34 | Bulgaria |
|  | PM2 | Karwendel | N 47.40 | E 11.50 | Austria |
|  | PM3 | Maiella | N 42.07 | E 14.10 | Italy |
|  | PM4 | East Carpathians | N 46.87 | E 25.70 | Romania |
|  | PM5 | Tatra Mountains | N 49.24 | E 20.00 | Poland |
|  | PM6 | Loven Tatra Mountains | N 49.50 | E 19.28 | Slovakia |
|  | PM7 | Zhabyak | N 43.11 | E 20.98 | Serbia |
|  | PM8 | Brenta | N 46.26 | E 10.95 | Italy |
|  | PM9 | Sudeten Range | N 50.76 | E 15.78 | Poland |
|  | PM10 | Southern Carpathian | N 45.11 | E 23.37 | Romania |
|  | PM11 | Oriental Pyrenees | N 42.43 | E 1.37 | Spain |
|  | PM12 | Kamnik-Savinj Alps | N 46.36 | E 14.54 | Slovenia |
|  |  |  |  |  |  |
| *Pinus cembra* | PC1 | Niederthai | N 47.13 | E 10.99 | Austria |
|  | PC2 | Oberems | N 46.45 | E 8.15 | Switzerland |
|  | PC3 | Grachen Hannigalp | N 46.34 | E 8.44 | Switzerland |
|  | PC4 | Tamangur-Schuls | N 47.14 | E 10.60 | Switzerland |
|  | PC5 | Nationalpark Hohe Tauern | N 46.95 | E 12.31 | Austria |
|  | PC6 | Bärnthal | N 47.03 | E 14.60 | Austria |
|  | PC7 | Lanersbach | N 47.15 | E 11.74 | Austria |
|  | PC8 | Schweizerischer Nationalpark | N 46.71 | E 10.29 | Switzerland |
|  | PC9 | Schöderberg | N 47.20 | E 14.08 | Austria |
|  | PC10 | Tamangur-Schuls | N 47.14 | E 10.60 | Switzerland |
|  | PC11 | Bükki National Park | N 48.06 | E 20.55 | Hungary |
|  | PC12 | High Tatra | N 49.16 | E 20.16 | Slovakia |

Table S2: Estimates of nucleotide diversity in the control genes for several site types.

| **Species** | **Parameters** | **All** ^c^ | **N-coding** ^c^ | **N-Syn** ^c^ | **Syn** ^c^ | **All silent**  ^c^ |
| --- | --- | --- | --- | --- | --- | --- |
| *A. alba* | Sites^a^ | **8000** | **1699** | **4892** | **1306** | **37.56%** |
|  | Segregating sites^a^ | **82** | **42** | **27** | **13** | **67.07%** |
|  | Watterson’s θ ^b^ | **0.0051** | **0.0027** | **0.0047** | **0.0061** |  |
|  |  | 0.0119 | 0.0088 | 0.0108 | 0.0145 |  |
|  | Nucleotide diversity (π) ^b^ | **0.0046** | **0.0023** | **0.0043** | **0.0051** |  |
|  |  | 0.0110 | 0.0071 | 0.0100 | 0.0130 |  |
|  |  |  |  |  |  |  |
| *L. decidua* | Sites^a^ | **8711** | **2091** | **5164** | **1372** | **39.75%** |
|  | Segregating sites^a^ | **42** | **0** | **23** | **19** | **45.23%** |
|  | Watterson’s θ ^b^ | **0.0037** | **0** | **0.0025** | **0.0092** |  |
|  |  | 0.0142 | 0 | 0.0115 | 0.0264 |  |
|  | Nucleotide diversity (π) ^b^ | **0.0043** | **0** | **0.0030** | **0.0102** |  |
|  |  | 0.0169 | 0 | 0.0114 | 0.0307 |  |
|  |  |  |  |  |  |  |
| *P. cembra* | Sites^a^ | **21644** | **4385** | **13357** | **3592** | **36.86%** |
|  | Segregating sites^a^ | **43** | **14** | **13** | **16** | **69.77%** |
|  | Watterson’s θ ^b^ | **0.0009** | **0.0005** | **0.0006** | **0.0019** |  |
|  |  | 0.0018 | 0.0017 | 0.0014 | 0.0052 |  |
|  | Nucleotide diversity (π) ^b^ | **0.0009** | **0.0005** | **0.0006** | **0.0020** |  |
|  |  | 0.0017 | 0.0016 | 0.0014 | 0.0053 |  |
|  |  |  |  |  |  |  |
| *P. mugo* | Sites^a^ | **22471** | **5530** | **13182** | **3547** | **40.39%** |
|  | Segregating sites^a^ | **176** | **57** | **54** | **65** | **69.32%** |
|  | Watterson’s θ ^b^ | **0.0051** | **0.0036** | **0.0025** | **0.0116** |  |
|  |  | 0.0110 | 0.0098 | 0.0073 | 0.0219 |  |
|  | Nucleotide diversity (π) ^b^ | **0.0050** | **0.0035** | **0.0024** | **0.0112** |  |
|  |  | 0.0113 | 0.0098 | 0.0077 | 0.0215 |  |

^a^ Numbers are the total number of sites and the total number of segregating size.

^b^ Numbers are the average across loci with the standard deviations are in regular type.

^c^ All, all sites; N-coding, non coding sites; N-Syn, non synonymous sites; Syn, synonymous sites; All silent, all silent sites.

Table S3: Estimates of the neutrality tests per gene set (candidate vs control) for each species.

| **Species** | **Gene set** | **D^a^** | **sd** | **H^a^** | **sd** | **EW^a^** | **sd** | **Zns^a^** | **sd** |
| --- | --- | --- | --- | --- | --- | --- | --- | --- | --- |
| *A. alba* | candidate | -0.13 | 0.96 | -0.13 | 1.14 | 0.74 | 0.26 | 0.71 | 0.33 |
|  | control | -0.44 | 0.19 | -0.34 | 0.38 | 0.71 | 0.23 | 0.60 | 0.39 |
| *L. decidua* | candidate | 1.02 | 0.13 | -0.26 | 0.28 | 0.74 | 0.26 | 0.85 | 0.28 |
|  | control | 0.02 | 0.28 | -0.28 | 0.39 | 0.81 | 0.27 | 0.69 | 0.34 |
| *P. cembra* | candidate | -0.02 | 0.26 | -0.48 | 0.32 | 0.81 | 0.22 | 0.85 | 0.25 |
|  | control | 0.09 | 0.22 | -0.64 | 0.29 | 0.86 | 0.21 | 0.92 | 0.19 |
| *P. mugo* | candidate | -0.21 | 0.23 | -0.28 | 0.35 | 0.61 | 0.23 | 0.66 | 0.35 |
|  | control | -0.42 | 0.19 | -0.09 | 0.33 | 0.63 | 0.25 | 0.59 | 0.37 |

**^a^** Numbers are average across genes with their standard deviation (D=Tajima’s D; H=Fay and Wu’s normalized H and EW= Ewens-Watterson’s F)

Table S4: List of the outliers from the standard neutral model (SNM) across the control genes.

| **Species** | **Gene** | **S ^c^** | **P_D_ ^a^** | **P_H_ ^a^** | **P_EW_ ^a^** | **P_Zns_ ^c^** | **DHEW-*P** ^b^** |
| --- | --- | --- | --- | --- | --- | --- | --- |
| *P. cembra* | 2_3465 | 3 | 0.0796 | 0.1337 | 0.903 | 1 | 0.17834 |
|  | 2_5724 | 2 | 0.1924 | 0.1349 | 0.7916 | 0.7831 | 0.26154 |
|  | 2_7852 | 2 | 0.109 | 0.0048 | 0.8125 | 1 | 0.31778 |
|  |  |  |  |  |  |  |  |
| *P. mugo* | 0_17206 | 4 | 0.0614 | 0.0003 | 0.9409 | 0.9397 | 0.11352 |
|  | 0_7171 | 3 | 0.1476 | 0.0029 | 0.8881 | 0.7799 | 0.15634 |
|  | 1_6493 | 4 | 0.0357 | 0.0282 | 0.9501 | 1 | 0.13516 |
|  | 2_8214 | 2 | 0.1209 | 0.0781 | 0.8119 | 1 | 0.29976 |
|  | UMN_1263 | 2 | 0.1922 | 0.126 | 0.7858 | 0.7742 | 0.26218 |

**^a^** The results of each test (D=Tajima’s D; H=Fay and Wu’s H and EW= Ewens-Watterson’s F) are presented as P-value.

**^b^** The critical P-values calculated with the compound DHEW test.

**^c^** The P value of Kelly Zns (P**_Zns_**) and the number of SNPs per locus (S).
